# Recurrent Vulvovaginal Candidiasis: a Dynamic Interkingdom Biofilm Disease of *Candida* and *Lactobacillus*

**DOI:** 10.1128/mSystems.00622-21

**Published:** 2021-08-10

**Authors:** Emily McKloud, Christopher Delaney, Leighann Sherry, Ryan Kean, Shanice Williams, Rebecca Metcalfe, Rachael Thomas, Riina Richardson, Konstantinos Gerasimidis, Christopher J. Nile, Craig Williams, Gordon Ramage

**Affiliations:** a School of Medicine, Dentistry and Nursing, College of Medical, Veterinary and Life Sciences, Glasgow, UK; b School of Life Sciences, Glasgow, UK; c Department of Biological and Biomedical Sciences, School of Health and Life Sciences, Glasgow Caledonian University, Glasgow, UK; d Sandyford Sexual Health Service, NHS Greater Glasgow and Clyde, Glasgow, UK; e Division of Infection, Immunity and Respiratory Medicine, Faculty of Biology, Medicine and Health, University of Manchester, Manchester, UK; f School of Dental Sciences, University of Newcastle, Newcastle upon Tyne, UK; Mayo Clinic

**Keywords:** antifungal resistance, biofilm, *Candida*, clinical, interkingdom, *Lactobacillus*, microbiome, vulvovaginal candidiasis

## Abstract

Despite the strikingly high worldwide prevalence of vulvovaginal candidiasis (VVC), treatment options for recurrent VVC (RVVC) remain limited, with many women experiencing failed clinical treatment with frontline azoles. Further, the cause of onset and recurrence of disease is largely unknown, with few studies identifying potential mechanisms of treatment failure. This study aimed to assess a panel of clinical samples from healthy women and those with RVVC to investigate the influence of *Candida*, the vaginal microbiome, and how their interaction influences disease pathology. 16S rRNA sequencing characterized disease by a reduction in specific health-associated *Lactobacillus* species, such as Lactobacillus crispatus, coupled with an increase in Lactobacillus iners. *In vitro* analysis showed that Candida albicans clinical isolates are capable of heterogeneous biofilm formation, and we found the presence of hyphae and C. albicans aggregates in vaginal lavage fluid. Additionally, the ability of *Lactobacillus* to inhibit C. albicans biofilm formation and biofilm-related gene expression was demonstrated. Using RNA sequencing technology, we were able to identify a possible mechanism by which L. crispatus may contribute to re-establishing a healthy vaginal environment through amino acid acquisition from C. albicans. This study highlights the potential formation and impact of *Candida* biofilms in RVVC. Additionally, it suggests that RVVC is not entirely due to an arbitrary switch in C. albicans from commensal to pathogen and that understanding interactions between this yeast and vaginal *Lactobacillus* species may be crucial to elucidating the cause of RVVC and developing appropriate therapies.

**IMPORTANCE** RVVC is a significant burden, both economically and for women's health, but its prevalence is poorly documented globally due to the levels of self-treatment. Identifying triggers for development and recurrence of VVC and the pathogenesis of the microbes involved could considerably improve prevention and treatment options for women with recurrent, azole-resistant cases. This study therefore aimed to examine the interkingdom dynamics from healthy women and those with RVVC using next-generation sequencing techniques and to further investigate the molecular interactions between C. albicans and L. crispatus in a relevant biofilm coculture system.

## INTRODUCTION

Fungal infections are becoming increasingly recognized as a substantial health burden on the global population. Over 1 billion people are estimated to suffer from fungal infections each year, resulting in over 1.5 million deaths (1). These infections are commonly associated with mucosal sites, such as the vagina, gut, and oral cavity. Vaginitis is estimated to account for up to 7% of all visits to gynecologists and for up to 10 million general practitioner (GP) appointments annually (2). Vulvovaginal candidiasis (VVC) is not a reportable disease and is often self-treated with over-the-counter antifungal agents; therefore, its exact prevalence and distribution are impossible to determine. VVC is reported as the second most common cause of vaginitis, and it is estimated that 75% of women will suffer from VVC in their childbearing years, with up to 140 million of these women developing recurrent VVC (RVVC), defined as ≥4 cases within 1 year (3, 4). These recurrent cases are debilitating, impact quality of life, and are associated with psychological stress, pruritis, and discomfort (5, 6). VVC is associated with various risk factors, including the use of antibiotics and contraceptives, new sexual partners, and allergic responses to *Candida* antigens; however, no distinguishable cause has been identified (7, 8).

The biofilm-forming yeast Candida albicans is reported as the predominant pathogen responsible for up to 90% of VVC infections (4). C. albicans is commonly isolated from the vagina as an asymptomatic commensal with a carriage rate of up to 33% in premenopausal women (9). Other non*-*albicans *Candida* (NAC) species, most commonly Candida glabrata, C. parapsilosis, C. dubliniensis and C. krusei, account for 10 to 20% of VVC infections and are associated with complicated VVC, which exhibits less severe symptoms than C. albicans VVC but higher recurrence rates (10, 11). C. albicans has been shown to form biofilms on vaginal mucosa both *ex vivo* and *in vivo* by identification of significant fungal load, biofilm architecture, and extracellular matrix using confocal and electron microscopy (12). This is a possible contributing factor to failed clinical treatment resulting in persistent and recurrent VVC. Despite the identification of *Candida* biofilms on vaginal mucosa, the predicted therapeutic challenge this presents in VVC is still disputed. Other studies challenge the hypothesis of vaginal biofilms and instead suggest other factors, such as germ tube formation and polymicrobial tissue invasion, as more critical features of VVC (13, 14).

The vaginal microbiome is somewhat unique in that a healthy microbiome is associated with a less diverse community of microbes, predominantly lactobacilli such as Lactobacillus crispatus, L. iners, L. gasseri, and L. jensenii (15). Dysbiosis of the vaginal microbiome leads to an increase in species diversity, with fewer lactobacilli and greater numbers of pathogenic anaerobes, such as *Gardnerella* and *Prevotella* (16, 17). In healthy women, lactic acid bacteria (LAB) are thought to be responsible for maintaining a homeostatic microbiome by inhibiting growth and adhesion of other microbes via the production of secreted metabolites, such as lactic acid, biosurfactants, bacteriocins, and H_2_O_2_. L. crispatus, a potent producer of both d- and l- isomers of lactic acid as well as H_2_O_2_, is a prevalent commensal of the healthy vaginal environment in various microbiome studies (18, 19). Additionally, it is known to secrete the l-lactic acid isomer that has been extensively studied for its ability to lower vaginal pH, elicit anti-inflammatory responses, and inhibit microbial colonization (20, 21).

A single dose of oral fluconazole is sufficient to treat sporadic C. albicans VVC in 80 to 90% of cases (22). Treatment of VVC caused by NAC is more complicated, requiring prolonged suppressive azole therapies, and is often unsuccessful. The efficacy of therapeutics such as amphotericin B and boric acid has been assessed for the treatment of RVVC. Both drugs when delivered intravaginally for 14 to 21 days were found to be effective in around 70% of patients (23, 24). *Candida* species, predominantly C. albicans, are known to form thick tenacious biofilms that dramatically increase tolerance to antifungal drugs commonly used in the treatment of VVC, such as fluconazole, miconazole, and flucytosine (25). Indeed, sessile cells have been shown to tolerate antifungal concentrations 1,000-fold greater than their planktonic counterparts (26). Therefore, an alternative therapeutic strategy for RVVC may be through microbiome replacement therapy in the form of *Lactobacillus* probiotics (27). Probiotic therapy involves the administration of live microorganisms, which directly results in a health benefit for the patient (28). Due to the diversity of lactobacilli within the vaginal microbiome, it is difficult to estimate which species would be most important to replace with probiotic therapy. One study evaluated Lactobacillus plantarum P17630 combined with the standard treatment of clotrimazole for 3 days and concluded a potential resolution of vaginal discomfort (29). The probiotic potential of secreted metabolites of *Lactobacillus* against C. albicans has been summarized, identifying gaps in our knowledge of fungal-*Lactobacillus* interactions that could lead to improved treatment options (30–33). Currently, no probiotic therapies have been approved for the treatment of VVC/RVVC.

Clearly, RVVC is a significant burden, both economically and for women’s health, and its prevalence is poorly documented globally due to the levels of self-treatment. Identifying triggers for development and recurrence of VVC and the pathogenesis of the microbes involved could considerably improve prevention and treatment options for women with recurrent, azole-resistant cases. This study therefore aimed to examine the interkingdom dynamics in healthy women and those with RVVC using next-generation sequencing techniques and to further investigate the molecular interactions between C. albicans and L. crispatus in a relevant biofilm coculture system.

## RESULTS

### Clinical and microbiological assessment of patient samples.

The mechanisms behind the shift in candidal species from commensal to pathogenic yeast seen in RVVC onset remain poorly understood. The cause of infection is likely multifactorial, and here, we aimed to assess whether fungal or bacterial load may influence disease by using samples collected from healthy individuals (*n* = 60) and women with RVVC (*n* = 40). Clinical assessment was used to confirm RVVC diagnosis, which was further validated by significantly increased interleukin 8 (IL-8) expression levels (*P* < 0.0001) (Table S1). Patient demographic data collected through questionnaires found that patients had suffered from RVVC for an average of 8 months, with an average of 1 month since they last received treatment. Although higher numbers of women with RVVC had culturable *Candida* than healthy women, quantitative PCR (qPCR) analysis revealed *Candida* to be detectable in all but 2 patients.

10.1128/mSystems.00622-21.9 TABLE S1 Patient demographics of women recruited for the study. Patients defined as having RVVC had presented with symptoms of VVC four or more times within 1 year. Download Table S1, DOCX file, 0.06 MB. © Crown copyright 2021. 2021 Crown https://creativecommons.org/licenses/by/4.0/ This content is distributed under the terms of the Creative Commons Attribution 4.0 International license.

**FIG 1:**
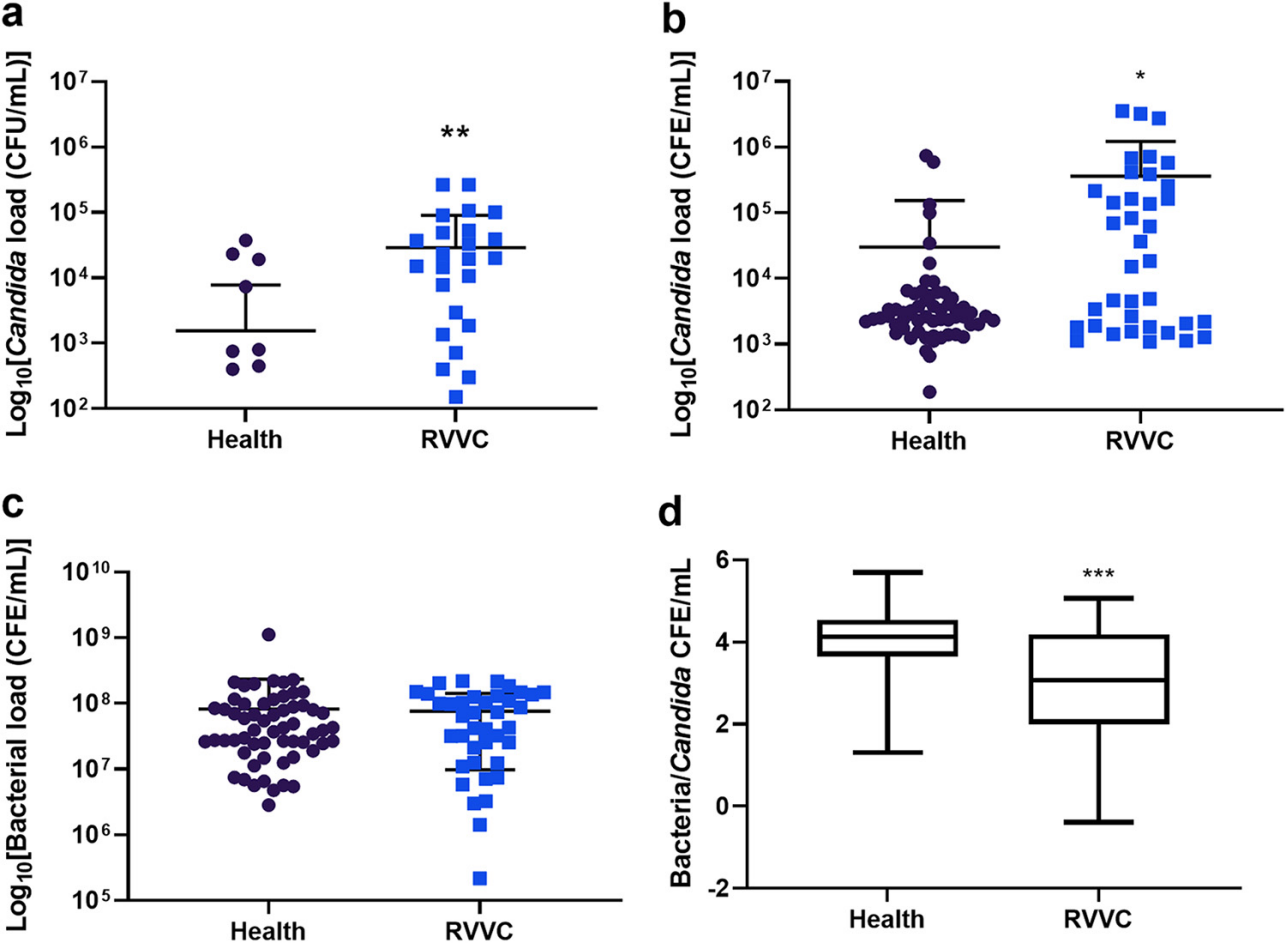
Fungal burden is elevated in women with RVVC while bacterial load remains unchanged. To assess fungal load, patient lavage was incubated on *Candida* Chromogenic agar plates and colonies counted after 48 h (a). For calculation of CFE/ml, the ITS region of *Candida* was amplified using genus-specific primers (b). Levels of bacteria DNA were also assessed molecularly by amplification of the 16S rRNA region (c). Correlation between bacterial and fungal burden was also observed (d). Data are means ± standard deviations (SD). Statistical significance was calculated using unpaired *t* tests with Welch’s correction, as data did not share equal standard deviations (*, *P* < 0.05; **, *P* < 0.01).

First, the levels of *Candida* present in the clinical samples were quantified by determining numbers of CFU per milliliter, in addition to total yeast and bacterial DNA quantified by qPCR colony-forming equivalents (CFE) per milliliter (Fig. 1). Quantities of yeast CFU/ml showed a significant increase in *Candida* load in samples, from approximately 2 × 10^3^ CFU/ml in healthy patients to 2.3 × 10^4^ CFU/ml in RVVC patients (*P* = 0.0079) (Fig. 1a). Similarly, when assessed by qPCR, a significant increase of around 1.3-log was observed in patients with RVVC, to 3.8 × 10^5^ CFE/ml (*P* = 0.024), as shown in Fig. 1b. These data suggest that increased levels of *Candida* could be a contributing factor to disease pathology or an indicator for VVC onset and subsequent recurrence. Additionally, *Candida* load was observed with respect to patient metadata. Although a slight reduction of between 0.5 and 1 log was observed in patients with disease for over 7 months, this was not significant (data not shown). An increase in fungal load from 1 × 10^4^ to 5 × 10^4^ CFU/ml was observed in women who had received treatment for RVVC longer than 1 month prior to sample collection (*P* = 0.021) (Fig. S2). Bacterial load was found to be comparable at ∼8 × 10^7^ CFE/ml between healthy women and those with RVVC (*P* = 0.779) (Fig. 1c). Finally, correlation between bacterial and fungal load found significantly higher levels of *Candida* DNA per CFE/ml of bacterial DNA present in RVVC compared with healthy controls (*P* = 0.0001) (Fig. 1d).

10.1128/mSystems.00622-21.2 FIG S2 Correlation of *Candida* load with treatment time. *Candida* loads, determined by CFU/ml (a and b) and CFE/ml (c and d), were analyzed with respect to the length of time since patients had received RVVC treatment. Data are means and SD; statistical analysis was performed using unpaired *t* tests (*, *P* < 0.05). Download FIG S2, TIF file, 0.09 MB. © Crown copyright 2021. 2021 Crown https://creativecommons.org/licenses/by/4.0/ This content is distributed under the terms of the Creative Commons Attribution 4.0 International license.

### Taxonomic classification of health and RVVC.

**FIG 2:**
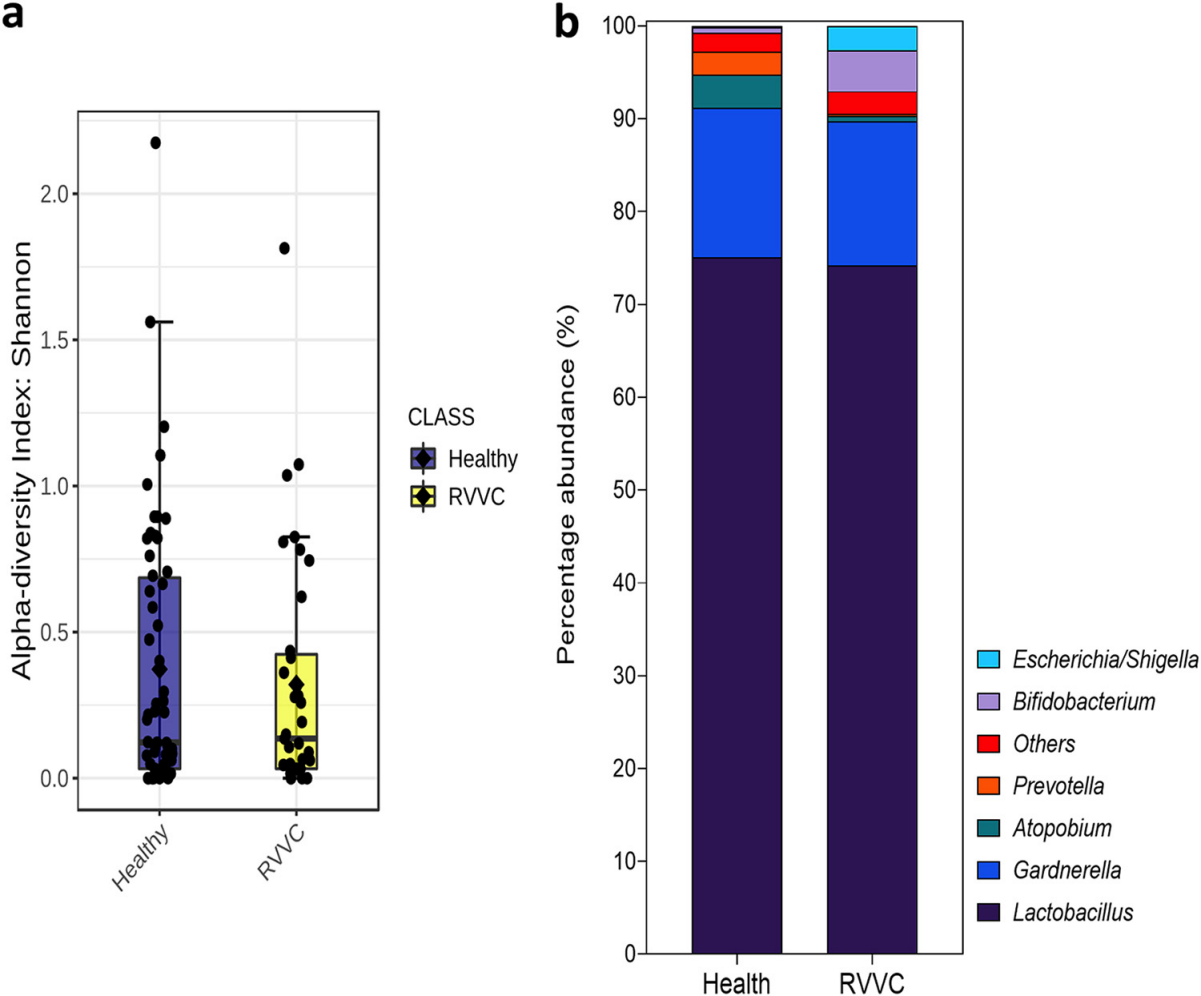
Bacterial genera present in healthy women and those with RVVC. DNA extracted from swab samples was used for 16S rRNA Illumina sequencing (*n* = 100). Bacterial diversity measured by Shannon index (a) and genus-level taxa identification and percentage abundance of microbial populations present (b) in health and RVVC.

**FIG 3:**
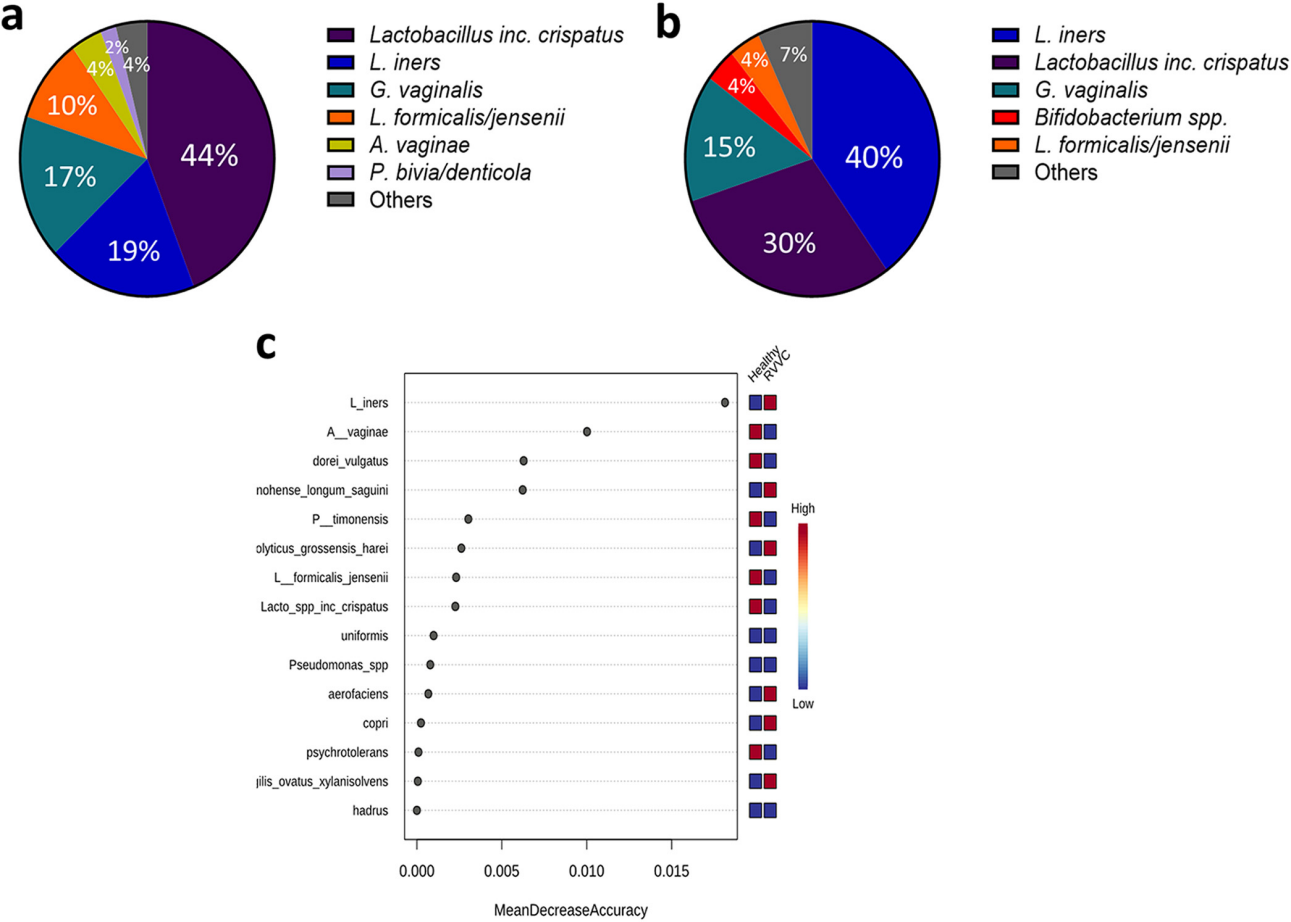
Hydrogen peroxide-producing lactobacillus strains are reduced during RVVC infection, resulting in an *L. iners*-dominated microbiome. Species-level identification of bacterial taxa present in healthy women (a) and those with RVVC (b). Random forest plot showing the most distinct species-level taxa present between healthy women and those with RVVC (c).

To determine the bacterial taxa present in healthy women and those with RVVC, we performed 16S rRNA sequencing on DNA extracted from swab samples (Fig. 2). At the genus level, a *Lactobacillus*-dominated environment, accounting for up to 75% of the microbiome, was observed in both cohorts, with similar levels of diversity and the vaginal anaerobes *Gardnerella a*nd *Prevotella* (Fig. 2a and b). *Atopobium* was found to be slightly higher in healthy patients and *Bifidobacterium* slightly lower. A limitation of our microbiome analysis was the inability to distinguish between some *Lactobacillus* species (including Lactobacillus acidophilus, L. casei, and L. gasseri). For this reason, these species are grouped as “*Lactobacillus* spp., including L. crispatus.” However, sequencing of a subset of samples using the Oxford Nanopore Technologies MinION sequencer confirmed accuracy to the species level (Fig. S3) (34). When viewed at the species level, although not significant following *P* value correction, subtle differences in bacterial taxa were observed between healthy controls and RVVC, particularly among *Lactobacillus* species (Fig. 3). Most notable was the reduction in levels of specific *Lactobacillus* species. This included L. jensenii and to a greater extent L. crispatus, which fell from 44% in healthy patients to 30% in those with RVVC (Fig. 3a and b). Interestingly, this reduction was coupled with an increase in *L. iners* from just 19% in healthy samples to 40% in RVVC. Additionally, when predicted using random forest plots, levels of *L. iners* were suggested to be the most distinct between samples from healthy women and those from women with RVVC (Fig. 3c).

10.1128/mSystems.00622-21.3 FIG S3 Specificity of genus and species-level identification is comparable across Illumina and Nanopore sequencing technologies. Bacterial taxonomy identification by Illumina to the genus (a) and species (c) levels compared with identification by Nanopore MinION identification to the genus (b) and species (d) levels. Download FIG S3, TIF file, 0.1 MB. © Crown copyright 2021. 2021 Crown https://creativecommons.org/licenses/by/4.0/ This content is distributed under the terms of the Creative Commons Attribution 4.0 International license.

**FIG 4:**
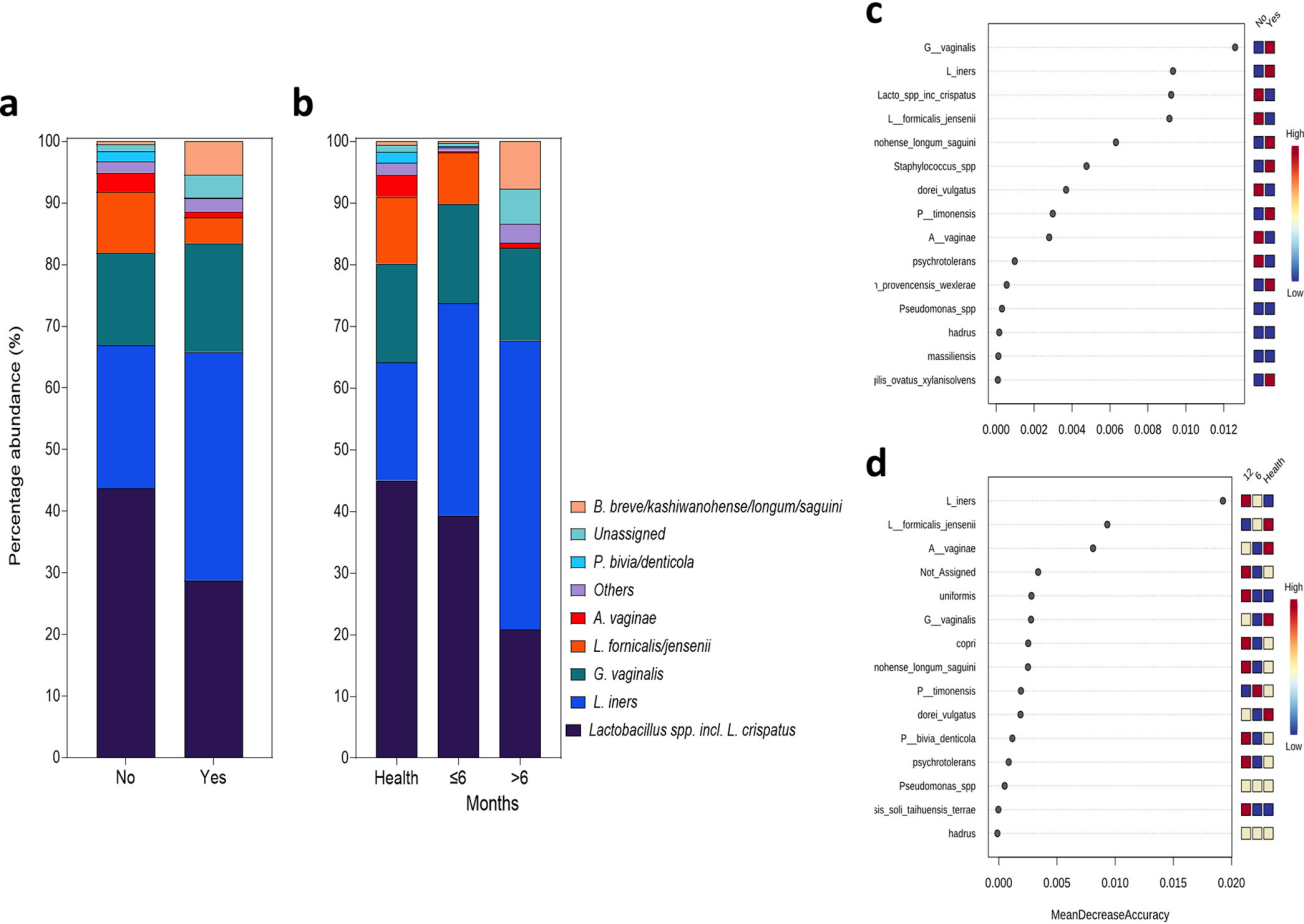
Species-level taxon abundance relative to patient metadata. Species-level bacterial taxa based on presence/absence of *Candida* (a) and length of time with disease (b). Random forest plots showing distinct bacterial taxa present in each analysis (c and d).

To investigate the influence of bacterial taxa further, microbial populations were observed with respect to patient metadata (Fig. 4). In patients with culturable *Candida* compared with those who were culture negative, a reduction in *Lactobacillus* species including L. crispatus from 44% to 29%, coupled with an increase in *L. iners* from 23% to 35%, was observed, similar to the RVVC profile (Fig. 4a). Further, *L. iners* was predicted to be the second most likely organism to define presence or absence of *Candida* using random forest analysis to identify important features (Fig. 4c). When observed with respect to the length of time patients had had RVVC, an intermediate profile between health and disease was observed in patients with disease for less than 6 months, showing a slight reduction in L. crispatus from 45% to 40% (Fig. 4c). Conversely, patients with disease for between 6 and 12 months had a profile similar to that seen in RVVC, with increased levels of *L. iners* from 19% to 44% (Fig. 4d). Additionally, bacterial communities present were observed with respect to contraceptives used at the time of sampling (Fig. S4). At the species level, a notable similarity in the composition of healthy patients and those using hormonal contraception, in contrast to patients using a contraceptive device or no contraception, was observed. The most important feature identified at the species level was predicted to be Atopobium vaginae. It is predicted that this organism is present in higher numbers in healthy patients and those using hormonal contraceptives and lower numbers in patients using either no contraception or contraceptive devices. Additionally, a contrasting pattern is shown for *L. iners*, which is predicted to be highest in patients using contraceptive devices. The microbiome was then observed with respect to how recently patients had received antifungal intervention to treat RVVC (Fig. S5). A gradual reduction in *Lactobacillus* species, including L. crispatus, was observed, from 44% in healthy samples to 34% and 23% in patients treated within a month and more than a month before sampling, respectively. Patients treated more than 1 month before sampling were predicted to have an intermediate profile between those of healthy women and women treated for RVVC more recently.

10.1128/mSystems.00622-21.4 FIG S4 Genus- and species-level taxon abundance relative to contraception used. Bacterial taxa present based on the contraception used by patients at the genus (a) and species (b) levels. Random forest plots showing bacterial taxa ranked by their contribution to each classification at the genus (c) and species (d) levels. Download FIG S4, TIF file, 0.2 MB. © Crown copyright 2021. 2021 Crown https://creativecommons.org/licenses/by/4.0/ This content is distributed under the terms of the Creative Commons Attribution 4.0 International license.

10.1128/mSystems.00622-21.5 FIG S5 Genus- and species-level taxon abundance relative to how recently patients had received antifungal treatment. Bacterial taxa present based on time since antifungal treatment (“1” represents ≤1 month and “4” represents >1 month prior to sampling) at the genus (a) and species (b) levels. Random forest plots showing bacterial taxa ranked by their contribution to each classification at the genus (c) and species (d) levels. Download FIG S5, TIF file, 0.2 MB. © Crown copyright 2021. 2021 Crown https://creativecommons.org/licenses/by/4.0/ This content is distributed under the terms of the Creative Commons Attribution 4.0 International license.

Finally, correlation between the vaginal microbiome and *Candida* load (CFE/ml) was observed with respect to patient metadata (Fig. S6). *Megasphaera*, often associated with vaginal dysbiosis, was found to be significantly positively correlated with increased fungal load (*P* < 0.05) (Fig. S6). Although not significant, genera such as *Dialister*, *Bacteroides*, and *Shuttleworthia* were correlated with higher fungal loads in RVVC. Further, *Lactobacillus* was significantly negatively correlated with *Candida* in healthy samples, and to a lesser extent in RVVC (*P* < 0.05). Although none were found to be significant, correlations observed with respect to recent treatment identified slight negative correlations between genera such as Streptococcus, *Gardnerella*, and *Lactobacillus* with increasing time since last RVVC intervention. Conversely, genera such as *Prevotella*, *Morganella*, and *Sneathia* were found to be positively correlated with increasing time since treatment. Streptococcus, *Prevotella*, and *Clostridium* were found to be positively correlated with increased RVVC episodes. However, genera such as *Shuttleworthia*, *Lactobacillus*, and *Atopobium* were found to be negatively correlated with increasing RVVC episodes.

10.1128/mSystems.00622-21.6 FIG S6 Correlation of fungal load with patient metadata. Heat map displaying correlations between bacterial genera present and *Candida* load (CFE/ml), recent treatment, and number of episodes of RVVC. Significance was measured using Pearson correlation tests (*, *P* < 0.05). Download FIG S6, TIF file, 0.1 MB. © Crown copyright 2021. 2021 Crown https://creativecommons.org/licenses/by/4.0/ This content is distributed under the terms of the Creative Commons Attribution 4.0 International license.

### Influence of *Candida* biofilm formation in RVVC.

**FIG 5:**
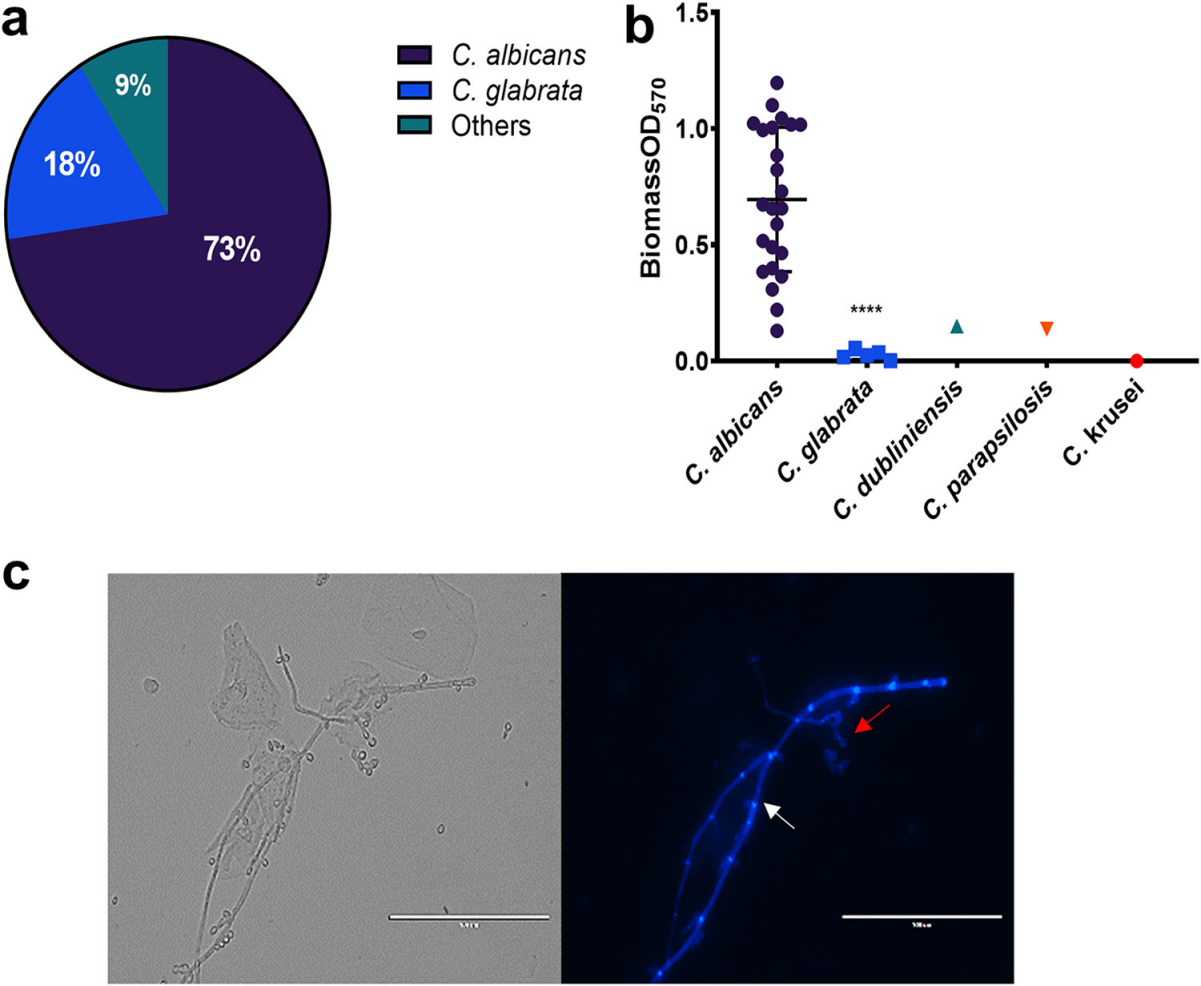
Vaginal *Candida* isolates from patients with RVVC are capable of heterogeneous biofilm formation. A total of 33 *Candida* clinical isolates were isolated from lavage samples. MALDI-TOF was used to identify each isolate and assess species distribution (“Others” comprises 1 isolate each of C. dubliniensis, C. parapsilosis, and C. krusei) (a). *Candida* isolates from healthy women (*n* = 9) and those with RVVC (*n* = 24) were assessed for biofilm-forming capabilities by crystal violet staining (b). Vaginal lavage was stained with calcofluor white (CFW) for 1 h at 37°C before imaging (c). Images are representative of C. albicans aggregates and hyphae observed in lavage fluid from a patient with an HBF isolate. White arrows represent pseudohyphal/hyphal formation, and red arrows depict cell aggregates. Data are means ± SD. Statistical analysis was performed using unpaired t-tests (****, *P* < 0.0001).

*Candida* clinical isolates were obtained from lavage samples by culture on *Candida* chromogenic agar. Following incubation, a total of 33 isolates were obtained, 9 from healthy individuals and 24 from patients with RVVC. Isolate identification was confirmed for all isolates using matrix-assisted laser desorption ionization–time of flight (MALDI-TOF) (Fig. 5a). Consistent with previous RVVC studies, C. albicans was found to account for 73% of the *Candida* species isolated within our patient subset. Other NAC species, more commonly associated with RVVC, accounted for the remaining 27%. Following identification, biofilm forming capabilities of clinical isolates was assessed (Fig. 5b). C. albicans was capable of forming dense biofilms with clear heterogeneity between isolates. Isolates were grouped as low biofilm formers (LBF) if the absorbance reading of their total biomass fell below the first quartile (<0.185). Similarly, high biofilm formers (HBF) were observed when total biomass was above the third quartile (>0.854). Intermediate biofilm formers (IBF) had biomass readings between these values. Minimal biomass was observed in NAC species. Additionally, when lavage fluid from an RVVC patient with a C. albicans isolate capable of dense biofilm formation was viewed microscopically, hyphal forms and bacterium-yeast aggregates could be clearly observed (Fig. 5c). To further investigate and validate that *Candida* biofilms are a defined clinical entity, expression levels of key biofilm-related genes were measured from patients’ vaginal lavage fluid. These data show detectable levels of expression of genes involved in hyphal morphogenesis, biofilm formation, and pathogenesis in *Candida* isolates present in patients with RVVC, including *HWP1*, *ECE1*, *ALS3*, and *SAP* (Table S2).

10.1128/mSystems.00622-21.10 TABLE S2 Key biofilm-related genes in C. albicans are expressed in lavage fluid from patients with RVVC. Samples from healthy (*n* = 10) and RVVC (*n* = 10) patients were assessed for expression of key biofilm-related genes in C. albicans. *, Expression levels are reported based on cycle threshold (*C_T_*) value: 3, high expression; 2, moderate expression; 1, low expression; 0, no expression. Download Table S2, DOCX file, 0.03 MB. © Crown copyright 2021. 2021 Crown https://creativecommons.org/licenses/by/4.0/ This content is distributed under the terms of the Creative Commons Attribution 4.0 International license.

### Antagonism of *Lactobacillus* and C. albicans.

**FIG 6:**
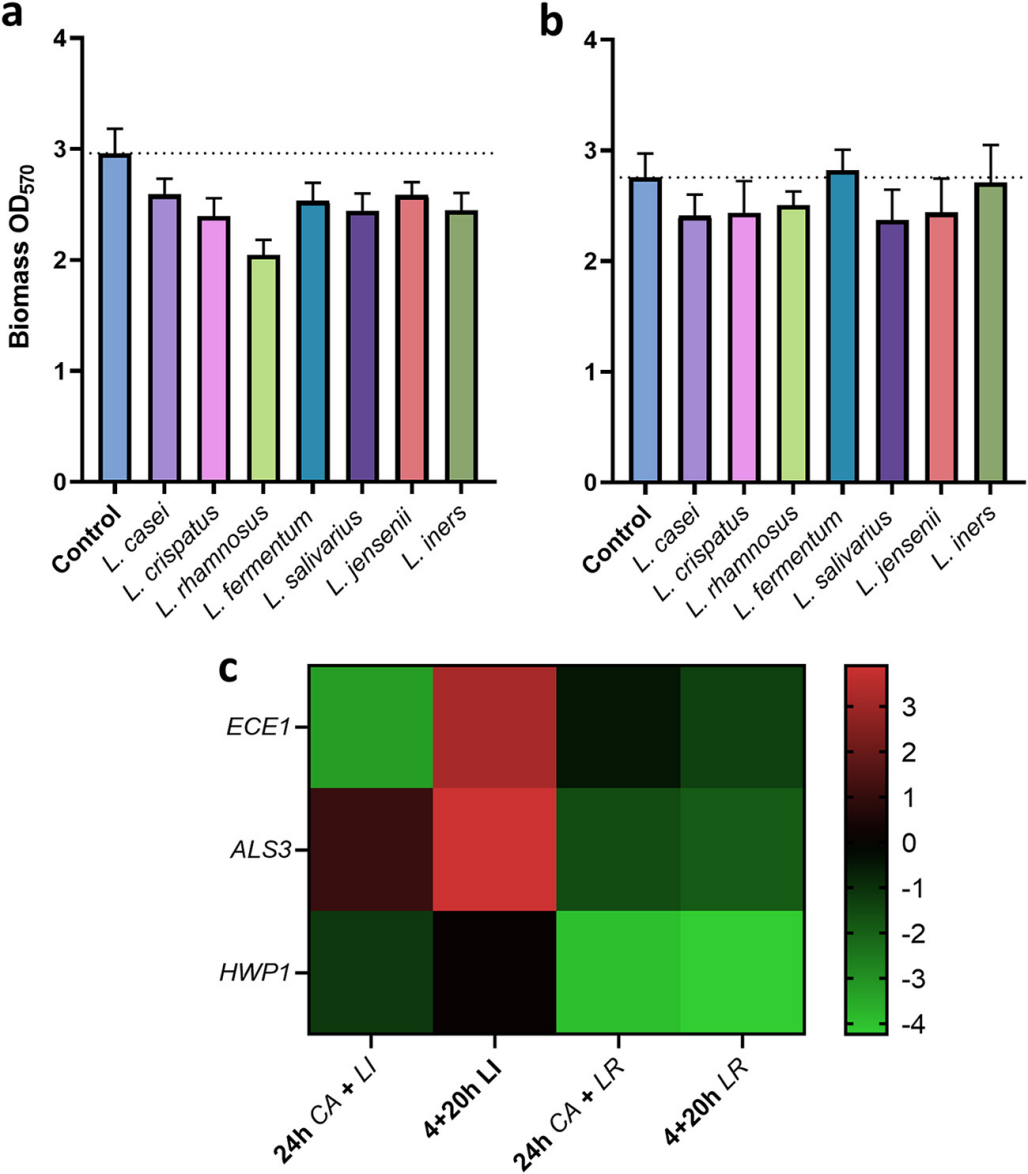
*Lactobacillus* species display antagonism with C. albicans *in vitro*. To observe inhibitory effects of *Lactobacillus* against C. albicans biofilm formation, C. albicans and a panel of *Lactobacillus* species were cocultured in THB/RPMI (1:1) medium in 5% CO_2_ for 24 h (a), or C. albicans was grown for 4 h prior to addition of *Lactobacillus* species for 20 h (b). C. albicans biofilm-associated gene expression was measured in the presence of L. rhamnosus, which is associated with health, and *L. iners*, which is hypothesized to indicate dysbiosis. The mean log fold change relative to single species C. albicans biofilms is shown (c). Data are means and SD.

Next, to assess the interactions between *Lactobacillus* species and C. albicans, 7 *Lactobacillus* species were selected and their effects on C. albicans biofilm formation in coculture observed (Fig. 6). When grown together for 24 h, a reduction in overall biomass was observed in all *Lactobacillus* species from an absorbance reading of 3.0 to approximately 2.5. This reduction was particularly prominent in Lactobacillus rhamnosus, where biomass was reduced to an absorbance value of 2.0 (Fig. 6a). However, when C. albicans was allowed to form a biofilm before addition of *Lactobacillus*, this effect was less pronounced and in some cases absent (Fig. 6b). To further analyze the biomass reduction, C. albicans biofilm-related gene expression was assessed following coculture with L. rhamnosus and *L. iners*. L. rhamnosus is often studied for its probiotic effect against C. albicans. Although *L. iners* is a vaginal commensal, it has not been found to inhibit fungal growth or biofilm formation. The two organisms differentially interacted with C. albicans, with L. rhamnosus downregulating all biofilm-related gene expression after incubation for 20 h and 24 h (Fig. 6c). Gene expression was reduced by ∼4-fold for *HWP1* and between 0.5- and 1-fold for *ALS3* and *ECE1* in both growth conditions. *L. iners* downregulated expression of *ECE1* and, to a lesser extent, *HWP1* after incubation for 24 h. However, when added to a preexisting C. albicans biofilm, *L. iners* resulted in upregulation of *ECE1* and *ALS3*.

**FIG 7:**
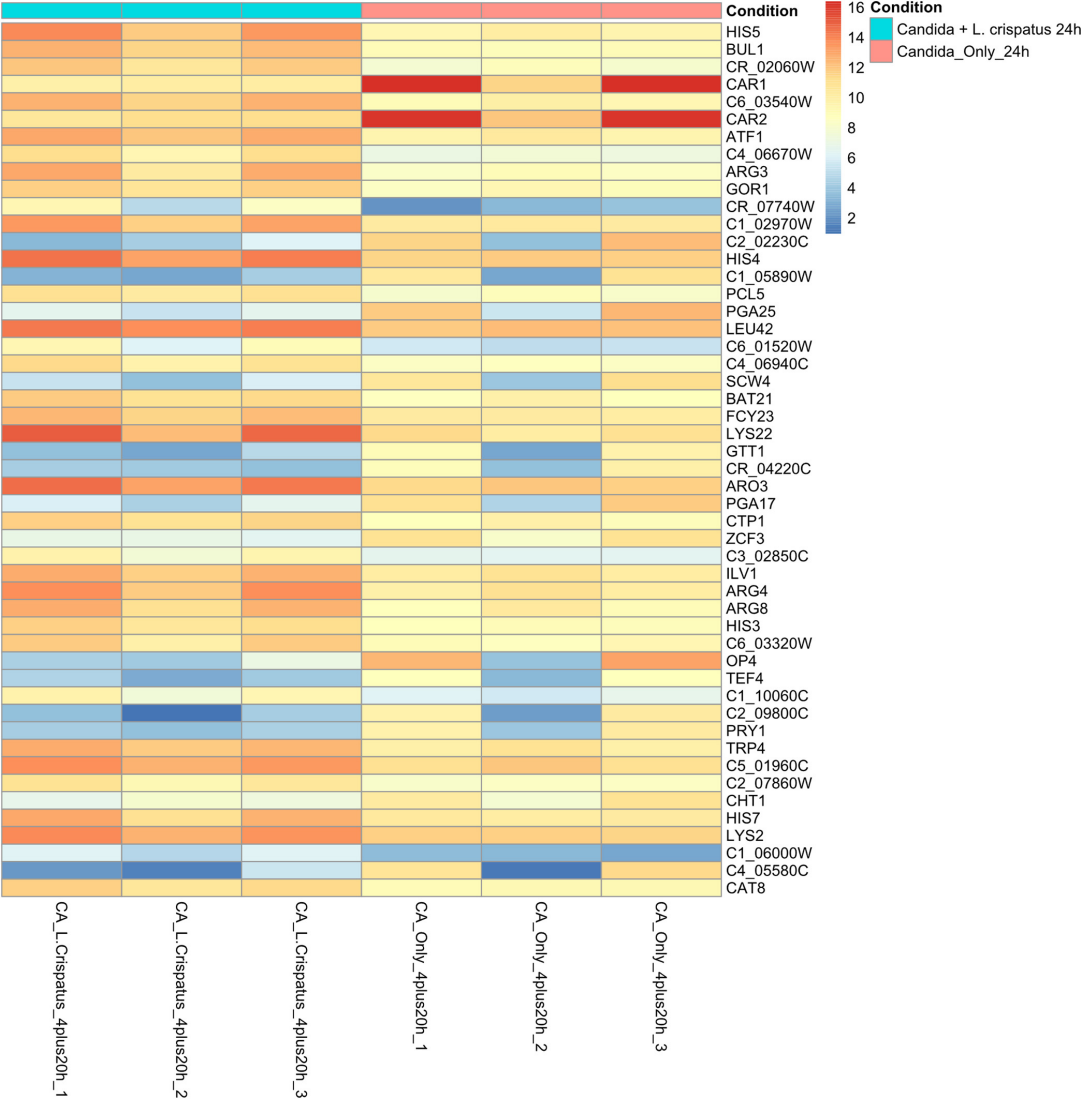
Differential expression analysis of C. albicans single-species and C. albicans–L. crispatus dual-species biofilms. Heat map displaying the top 50 significantly differentially expressed genes in C. albicans between single- and dual-species 24-h biofilms (*P* < 0.05).

**FIG 8:**
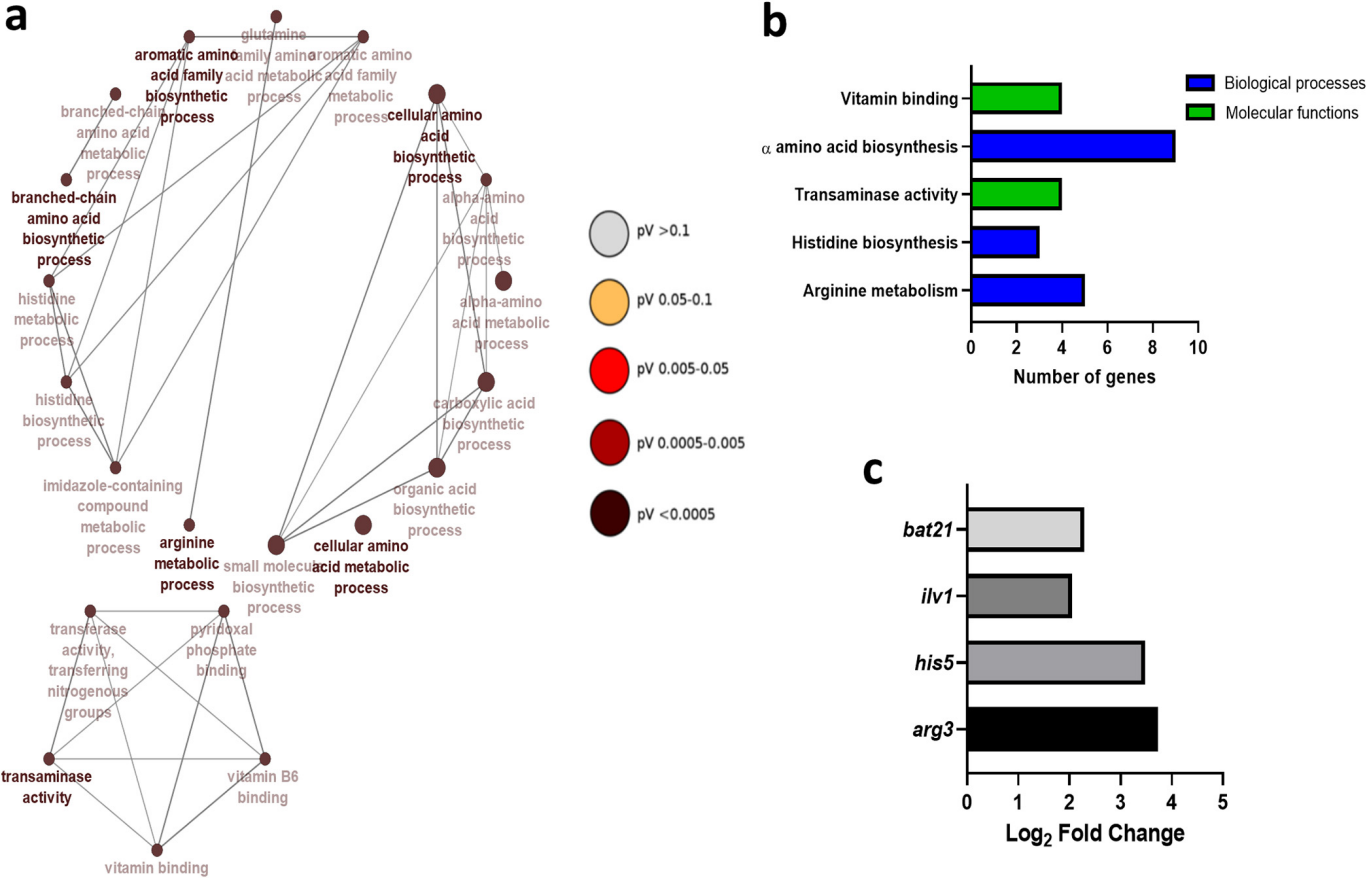
Gene networks of gene ontology (GO) terms and upregulated genes in dual-species biofilms. Constructed gene networks of GO terms in 24-h biofilms with L. crispatus (a). Important biological, cellular, and molecular functions in dual-species biofilms with L. crispatus (b). Log_2_ fold change of key gene expression in C. albicans from single- to dual-species biofilms with L. crispatus (c). Nodes are colored by significance. All GO terms have an adjusted *P* value of <0.05. Networks were created using ClueGO.

To further understand how antagonistic features of *Lactobacillus* impact C. albicans, we utilized transcriptome sequencing (RNA-Seq) of C. albicans following coculture with L. crispatus. L. crispatus was selected for RNA sequencing analysis, as opposed to L. rhamnosus, as it is not a commensal of the vaginal environment and is capable of only transient colonization. Further, L. crispatus is one of the most prevalent *Lactobacillus* species in the vaginal microbiome, and its antagonism toward C. albicans has been demonstrated in previous studies (31–33, 35). Initially, multivariate analysis by principal-component analysis (PCA) was unable to identify distinct clusters of gene expression in early dual-species biofilms (Fig. S8). Subsequently, there was no significant up- or downregulation in early dual-species biofilm transcripts compared to single-species controls (*P* > 0.05). For this reason, further analysis compared expression in mature 24-h single- and dual-species biofilms only. Heat map analysis of normalized log_2_ fold change in gene expression between single- and dual-species 24-h biofilms with L. crispatus revealed upregulation of many genes involved in amino acid biosynthesis and breakdown in dual-species biofilms (*ARG8*, *ILV1*, and *HIS5*) (Fig. 7). Interestingly, the *PRY1* gene, which codes for a secreted protein associated with virulence in the presence of lactate in C. albicans, was found to be downregulated in dual-species biofilms (log_2_ fold change = −5.35). A list of some of the key genes and their functions is presented in Table 1. Gene ontology (GO) term analysis was utilized to determine the functionality of differentially expressed genes in single- and dual-species 24-h biofilms. Genes expressed in L. crispatus dual-species biofilms were mainly responsible for amino acid biosynthesis and some metabolic and transaminase activity (Fig. 8a and b). Despite upregulation of these amino acid biosynthesis and metabolism pathways in C. albicans, expression of *BAT21* and *ILV1* in dual-species biofilms suggests that C. albicans was in a state of amino acid starvation (Fig. 8c).

**TABLE 1:** Upregulated genes in 24-h dual-species biofilm associated with amino acid biosynthesis and/or breakdown

Gene name	Function	Log_2_ fold change
*arg3*	Arginine and citrulline biosynthesis	3.72897
*his5*	Histidine biosynthesis	3.474415
*lys22*	Lysine biosynthesis	3.469013
*bul1*	Protein ubiquitination	3.254671
*arg4*	Arginine biosynthesis/metabolism	2.810158
*atf1*	*N*-Acetyltransferase activity	2.704281
*arg8*	Arginine biosynthesis/metabolism	2.695885
*aro3*	Phenylalanine, tyrosine, and tryptophan biosynthesis	2.411573
*his4*	Histidine, purine, and pyrimidine biosynthesis	2.352654
*his3*	Histidine, purine, and pyrimidine biosynthesis	2.340446
*bat21*	Leucine, valine, and isoleucine biosynthesis/breakdown	2.284677
*ilv1*	Leucine, valine, and isoleucine biosynthesis, threonine breakdown	2.049845
*leu42*	Leucine, valine, and isoleucine biosynthesis	1.945496

10.1128/mSystems.00622-21.7 FIG S7 Monitoring pH levels throughout C. albicans and L. crispatus coculture for transcriptome analysis. Overnight cultures of C. albicans and L. crispatus were standardized to 1 × 10^6^ and 1 × 10^7^, respectively. *Candida* biofilms were grown for 4 h before the addition of L. crispatus for 2, 4, or 20 h. After incubation, medium was removed, and pH levels of medium and single- and dual-species biofilms were measured. Experiments were carried out in triplicate, on three separate occasions. Data are means + SD; statistical significance was measured using *t* tests or one-way analysis of variance (ANOVA) (*, *P* < 0.05; #, *P* < 0.0001). Download FIG S7, TIF file, 0.07 MB. © Crown copyright 2021. 2021 Crown https://creativecommons.org/licenses/by/4.0/ This content is distributed under the terms of the Creative Commons Attribution 4.0 International license.

10.1128/mSystems.00622-21.8 FIG S8 Principal-component analysis (PCA) groups samples based on variance in expression. PCA analysis of differential gene expression in single- and dual-species C. albicans and L. crispatus biofilms. Download FIG S8, TIF file, 0.08 MB. © Crown copyright 2021. 2021 Crown https://creativecommons.org/licenses/by/4.0/ This content is distributed under the terms of the Creative Commons Attribution 4.0 International license.

**FIG 9:**
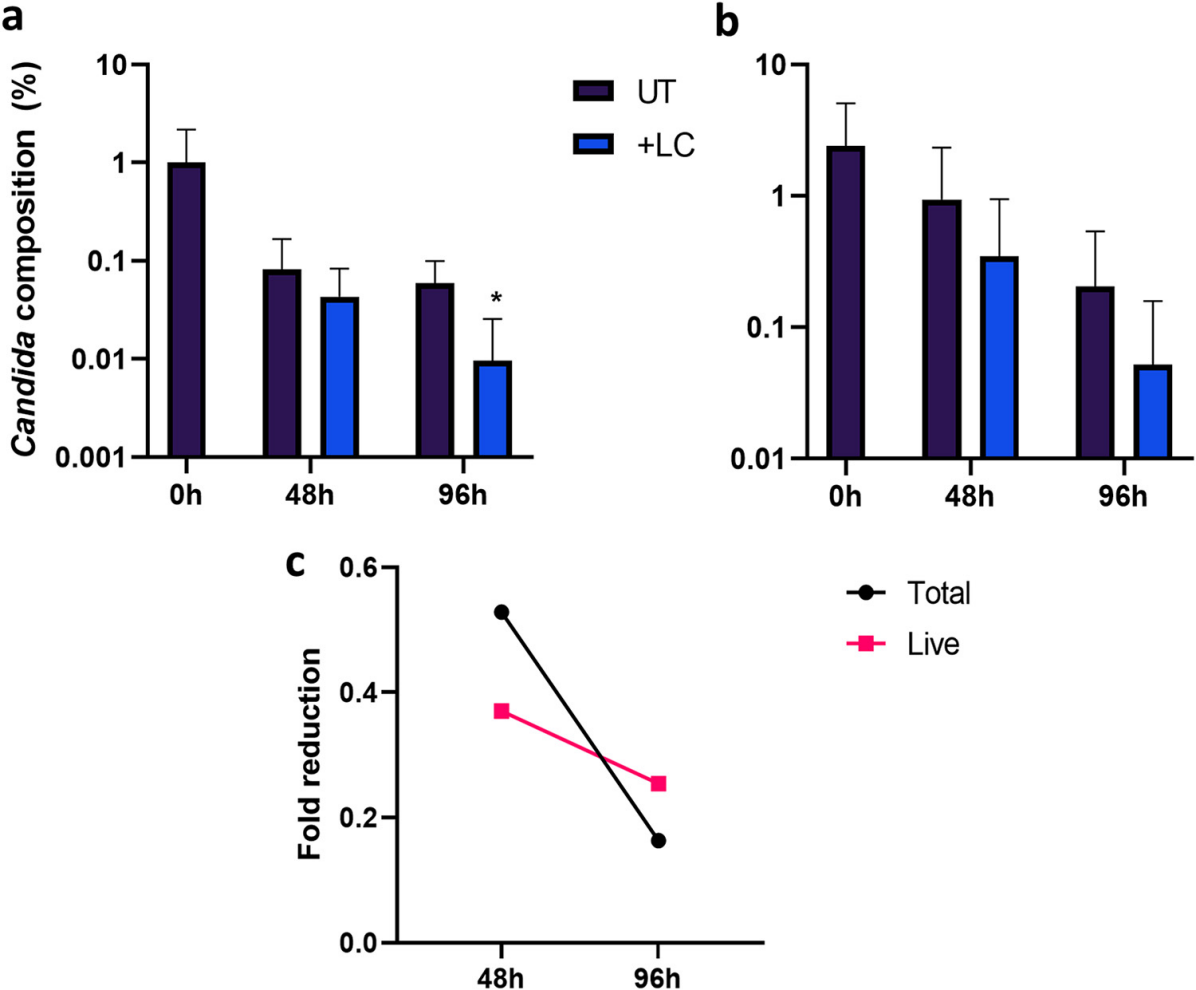
Twice-daily addition of L. crispatus reduces C. albicans load within a complex biofilm model *in vitro.* The potential probiotic properties of L. crispatus against C. albicans were assessed in a 11-species biofilm model treated twice daily with L. crispatus. Live/dead qPCR allowed quantification of percentage composition of total (a) and live (b) C. albicans DNA within the biofilm. Average fold change in the C. albicans percentage composition from the untreated 11-species biofilm is also shown (c). Data are means and SD. Statistical analysis was performed using paired *t* tests comparing raw CFE values (*, *P* < 0.05).

Given the antagonism observed between the two organisms, we next aimed to investigate the *in vitro* probiotic potential of L. crispatus against C. albicans infection in a complex biofilm model (Fig. 9). After 2 consecutive days of probiotic treatment, a slight reduction in total and live C. albicans composition within the biofilm was observed; however, this was not significant (*P* = 0.55 and *P* = 0.16, respectively) (Fig. 9a and b). Following a 4-day treatment regimen with L. crispatus, total C. albicans composition decreased (*P* < 0.05), with reduced levels of live fungal DNA. When the total fold reduction in C. albicans from untreated biofilms was assessed, the greatest probiotic effect was observed at 48 h posttreatment (Fig. 9c).

## DISCUSSION

Unlike systemic and oral candidiasis, RVVC affects immunocompetent women with an incidence rate of up to 8% in women of child-bearing age; hence, it is the most prevalent human infection caused by the pathogenic yeast, with approximately 140 million cases annually (36). Despite this, the disease remains largely understudied in the field of women’s health. A better understanding of RVVC at a molecular level may be crucial in unearthing potential targets for much-desired therapeutics. This study aimed to assess a panel of 100 clinical isolates to investigate fungal influence and changes in bacterial communities and to use an RNA sequencing approach to analyze potential interkingdom interactions contributing to pathology.

### Clinical and microbiological assessment of patient samples.

The estimated vaginal commensal carriage rate of *Candida* is 33% (9). We observed the presence of *Candida* both by culture and by qPCR and found that 15% and 60% of samples from healthy women and those with RVVC had culturable *Candida*, respectively, and all but 2 samples were positive by qPCR. As expected, levels of the inflammatory biomarker IL-8 and *Candida* DNA were found to be significantly higher in patients with RVVC, confirmatory of clinical diagnosis. It is important to note that these clinical samples are from women regularly attending sexual health clinics, and those who have suffered from RVVC for longer may be more likely to be receiving antifungal treatment. This should be considered when interpreting *Candida* CFE/ml data in this study, as untreated RVVC may present differently.

### Taxonomic classification of health and RVVC.

Unlike the vaginal microbiome during bacterial vaginosis (BV), the microbial communities present during VVC have been shown to be similar to those present in healthy women at the phylum and genus levels (37, 38). Our study confirms these findings, as we report a *Lactobacillus*-dominated population with vaginal anaerobes, including *Gardnerella*, *Prevotella*, and *Atopobium*, with no significant differences in diversity or composition between the two cohorts at the genus level. This suggests that the functional capacity of the bacterial species found in healthy women and those with RVVC may play a more crucial role in pathology. We observed a reduction in specific *Lactobacillus* species, including those associated with maintaining health due to their ability to produce l-lactic acid and H_2_O_2_, such as L. crispatus and L. jensenii. This reduction coupled with an increase of *L. iners* has been shown previously and is thought to be indicative of vaginal dysbiosis (37). We report that this loss of health-associated *Lactobacillus* species and increase of *L. iners* is also seen specifically in patients with positive *Candida* cultures and those who have suffered from RVVC for >6 months.

We show an *L. iners*-dominated microbiome in women using contraceptive devices or no contraception, which suggests that hormonal contraceptives may be less dysbiotic to the vaginal flora or have the potential to maintain a more health-like microbiome than contraceptive devices. Additionally, recent antifungal use revealed a microbial profile similar to those of healthy patients, suggesting that, although somewhat effective, the current antifungal treatments for RVVC do not alter the microbiome to a sufficient level to reintroduce a health-like state. This is evident with the microbiome reverting to favor a disease-like environment after less than 1 month. This is similar to results found by other authors, who reported a transition from disease to an intermediate state following fluconazole treatment (16). Although not associated with vaginal dysbiosis, the genus *Shuttleworthia* was positively correlated with *Candida* load and increased time between treatments and is often observed in high levels in vaginal microbiome studies (39). Regardless of disease status, *Lactobacillus* was negatively correlated with the presence of *Candida* as well as increased numbers of episodes and time between treatments, indicating the potential protection from *Candida* overgrowth provided by vaginal lactobacilli. These findings may be important for future studies investigating RVVC. It is hypothesized that changes within *Candida* allow it to switch from asymptomatic commensal to pathogenic yeast. It may now be more important to study RVVC with a specific focus on the microbes present during RVVC, specifically *Lactobacillus*, and interkingdom interactions which may influence this pathogenic switch in *Candida*.

### Influence of *Candida* biofilm formation in RVVC.

Consistent with epidemiological reports, we observed C. albicans to be the most common isolate identified, followed by C. glabrata. Though C. albicans has been shown to form biofilms on vaginal mucosa and *Candida* clinical isolates have been shown to form biofilms *in vitro*, the presence of biofilms during RVVC infection is still disputed (37). Despite evidence to the contrary, our study has shown heterogeneous biofilm formation in C. albicans RVVC isolates and shows clear visualization of C. albicans hyphae and aggregates in lavage fluid from a patient with RVVC. Moreover, transcripts from genes associated with biofilm formation were readily detected from vaginal lavage fluid. Therefore, *Candida* biofilm formation within the vagina should not be discounted as a potential cause of failed clinical treatment and subsequent recurrence of disease. We also show the capacity of various *Lactobacillus* species to inhibit C. albicans biofilm formation when cocultured. L. rhamnosus has been studied extensively for its potential as a probiotic and has been shown to prevent adhesion of C. albicans to mucosal surfaces (39). Here, we show the ability of L. rhamnosus to downregulate C. albicans biofilm-related gene expression. In contrast, *L. iners* results in upregulation of both *ALS3* and *ECE1*. This is consistent with previous findings suggesting that *L. iners* may be indicative of a dysbiotic vaginal environment and should not be considered as a probiotic intervention for C. albicans infections (40).

### Antagonism of *Lactobacillus* and C. albicans.

In our transcriptomic analyses we aimed to examine mechanisms of reported antagonism between C. albicans and L. crispatus. It has previously been shown in *Lactobacillus* clinical isolates that secreted metabolites can remain at low levels from 72 or 120 h of incubation, which may account for the delay in interaction (41). The *in vivo* vaginal pH is thought to be <4.5; however, in this dual-species model, the pH was sustained between 6 and 7. It is possible that in the *in vivo* setting, the lower environmental pH could affect C. albicans gene expression. Nonetheless, the stability of the neutral pH throughout this experiment dictates that any transcriptomic changes observed are not due to acidification of the medium and are a result of interactions between C. albicans and L. crispatus.

Transaminase activity is primarily associated with α-amino acid breakdown and biosynthesis. It has been shown in a model of Saccharomyces cerevisiae, L. lactis, and *L. plantarum* that in nitrogen-rich environments, yeasts secrete an array of metabolites, primarily amino acids, thereby facilitating the growth of lactobacilli (42, 43). The upregulation of various amino acid biosynthesis processes in our study may be attributed to this nutrient cross-feeding. As opposed to a convenient coincidence, this may be a deliberate process by which L. crispatus is able to drive synthesis of amino acids in C. albicans, as well as suppressing arginine breakdown, to facilitate its own metabolism and growth. The upregulation of the amino acid starvation indicators *bat21* and *ilv1* in dual-species biofilms shows that despite the upregulation of various amino acid biosynthesis processes, C. albicans is unable to utilize them. This may be a contributing factor in the process by which L. crispatus is able to outcompete *Candida* during VVC and potentially re-establish a healthy microbiome. Furthermore, the *pry1* gene, encoding a secreted glycoprotein associated with virulence and sterol binding in the presence of lactate, was found to be downregulated in dual-species biofilms (44). Thus, L. crispatus may possess a mechanism by which it can suppress lactate-associated virulence in C. albicans. Species-specific differential transcriptomic profiles in vaginal candidiasis have been demonstrated, and therefore, interactions between *Lactobacillus* and other prevalent *Candida* species should be considered (44). These data collectively show a potentially probiotic effect of L. crispatus against C. albicans in nonacidic environments. This antagonism is governed by the overproduction of amino acids from C. albicans, which may facilitate restoration of a healthy microbiome through lactobacillus proliferation.

Finally, we investigated the potential probiotic effect of L. crispatus against C. albicans within a complex biofilm model. Although studies have assessed the inhibitory effect of L. crispatus against C. albicans monospecies biofilms (33, 45, 46), to our knowledge, this is the first report of L. crispatus reducing C. albicans composition within a polymicrobial biofilm model *in vitro*. Our data support other studies confirming the probiotic potential of L. crispatus against C. albicans biofilm infections.

This study assessed the vaginal microbiome only of women in the United Kingdom; results may differ based on geographical prevalence of different *Candida* and *Lactobacillus* species. Additionally, a larger sample set may allow for statistically significant differences in the microbiome of women with VVC and healthy controls. It may be useful for future studies to observe transcriptional changes of C. albicans with other health-associated vaginal *Lactobacillus* species, such as L. jensenii and L. gasseri.

In conclusion, *Candida* burden is increased in women with RVVC compared to asymptomatic controls and continues to increase following antifungal intervention due to resistant communities. The reduction of protective H_2_O_2_-producing *Lactobacillus* species during RVVC is exacerbated in women who have RVVC for longer than 6 months, those who have culturable *Candida*, those who use no contraception or a contraceptive device, and those who are treated less frequently. Additionally, demonstration of biofilm-forming capabilities and imaging of C. albicans aggregates in patient lavage fluid, as well as the expression of C. albicans biofilm-related genes, suggest that there is a high probability of *Candida* biofilm formation in the vagina during RVVC. The subsequent increased antifungal tolerance of these biofilms may allow *Candida* to persist within the vagina and cause recurrence of VVC. Our study suggests that it may be important to view RVVC as a result of fluctuation in antagonistic interkingdom interactions between *Candida* and *Lactobacillus*, potentially as a result of *Candida* biofilm formation. Additionally, we show for the first time the potential of L. crispatus to re-establish a healthy vaginal environment through altered gene expression in C. albicans and the ability to reduce fungal composition in a complex biofilm model. Lactobacilli are key protective organisms in the healthy vaginal microbiome, and their antagonistic effect on *Candida* is of clinical significance. As demonstrated within our clinical study, there was a decrease in levels of protective *Lactobacillus* species in RVVC patients. The decrease of levels of these species also inversely correlated with an increase in fungal load. Further understanding of this antagonism offers potential avenues for improving women’s health through probiotic/prebiotic regimens. This interaction could prove impactful in the development of novel mechanisms for antifungal treatments, and therefore, further studies of this interaction are required.

## MATERIALS AND METHODS

### Patient recruitment and collection of clinical samples.

One hundred women aged 18 and over attending Glasgow Sandyford Sexual Health Clinic were enrolled in the study. Patient recruitment and obtaining of written informed consent were carried out at the clinic from prospective participants after they had read the patient information sheet. If the patient chose to participate, a questionnaire was completed and given to the clinician in charge of the study (R.M.). Patients with current, confirmed symptomatic RVVC (*n* = 40) were recruited as well as asymptomatic women attending the clinic for contraceptive intrauterine device (IUD) implantation, acting as a healthy cohort (*n* = 60). This study was granted ethical approval by the Sheffield Research Ethics Committee (16/YH/0310). Patients were excluded from the study if they had prior or active bacterial vaginosis, were pregnant, immunosuppressed, menstruating, or menopausal, or had taken antibiotics/antifungals within 7 days prior to sampling. From each patient, one high vaginal swab (HVS) and one cervicovaginal lavage fluid sample (CVL) were collected. A graphical representation of sample collection and processing can be found in Fig. S1.

10.1128/mSystems.00622-21.1 FIG S1 Illustrative representation of RVVC clinical study sample collection, sample processing, and transcriptome study design. High-vaginal swab and cervicovaginal lavage samples were collected from healthy women and those with RVVC attending their GP clinics. Lavage samples were used for *Candida* clinical isolate isolation and identification. DNA was extracted from swab samples to quantify fungal and bacterial load (a). Experimental (b) and bioinformatics (c) pipeline for C. albicans and L. crispatus coculture biofilm formation, RNA extraction, transcriptome sequencing, and expression analysis. Created with BioRender. Download FIG S1, TIF file, 0.2 MB. © Crown copyright 2021. 2021 Crown https://creativecommons.org/licenses/by/4.0/ This content is distributed under the terms of the Creative Commons Attribution 4.0 International license.

### Detection of inflammatory biomarkers in CVL fluid.

CVL fluid supernatants were recovered by centrifugation. Levels of IL-8 were measured by enzyme-linked immunosorbent assay (ELISA) (Invitrogen, Paisley, UK), following the manufacturer’s instructions. CVL fluid was diluted 1:2, and absorbances were measured using a spectrophotometer at 450 nm and 570 nm.

### Quantification of microbial load by quantitative PCR.

HVS samples were used to extract DNA for quantitative PCR (qPCR) analysis of *Candida*/bacterial burden. DNA was extracted using the QIAamp DNA minikit, per the manufacturer’s instructions (Qiagen, Crawley, UK), and qPCR was used to quantify fungal and bacterial load in each sample. Primers specific to the conserved *Candida* internal transcribed spacer (ITS) rRNA gene (47) were used to determine *Candida* load. For bacterial load, primers and probe specific to the 16S rRNA gene were used (48, 49). Primer sequences can be found at https://www.glasgowbiofilms.co.uk/. The total qPCR volume was 20 μl, with 1 μl of extracted DNA, 500 μM forward/reverse primers, and UV-treated RNase-free H_2_O. For 16S, 250 μM probe and 2× TaqMan universal PCR master mix (Thermo Scientific, Loughborough, UK) was used; 2× Fast SYBR green PCR master mix (Thermo Scientific, Loughborough, UK) was used for ITS primers. qPCR was carried out using a Step-One Plus real-time PCR machine (Life Technologies, Paisley, UK) with the following thermal conditions: an activation step of 50°C for 2 min and 95°C for 10 min followed by 40 cycles of 95°C for 10 s to denature and annealing at 60°C for 30 s. Standard curves constructed from serially diluted DNA of C. albicans SC5314 and Escherichia coli K-12 were used to extrapolate *Candida* and bacterial colony-forming equivalents (CFE) per milliliter, respectively, as described previously (50).

### Preparation of 16S amplicon libraries for Illumina sequencing.

To observe bacterial populations in the samples, 16S rRNA sequencing was performed. Briefly, previously extracted HVS DNA was used to sequence the 16S rRNA V4 region using the Illumina MiSeq sequencing platform (Edinburgh Genomics) using 2 × 250-bp paired-end reads. Amplification of the V4 region was achieved using fusion Golay adaptors barcoded on the reverse strand as described previously (51) using the universal primer 515F (5′-TATGGTAATTGTGTGCCAGCMGCCGCGGTAA-3′) and reverse primer 806R (5′-AGTCAGTCAGCCGGACTACHVGGGTWTCTAAT-3′). Quality control (QC) of reads was performed within the R package DADA2 (v1.14.1). Reads were filtered and trimmed using standard error rates before reads were denoised and merged and chimeric sequences removed. Amplicon sequence variants (ASV) were annotated by the Silva 132 16S rRNA database with an exact match to the ASV. ASV and taxonomic assignments were utilized for downstream data analysis.

### Identification and biofilm screening of *Candida* species from cervicovaginal lavage fluid.

Lavage fluid samples were screened for presence and identification of *Candida* species using Colorex *Candida* chromogenic agar (E&O Laboratories Ltd., Bonnybridge, UK) and matrix-assisted laser desorption ionization–time of flight (MALDI-TOF) mass spectrometry. For culture identification, 20 μl of CVL samples was spread across the surface of a chromogenic agar plate before 48 h of incubation at 30°C. The color of the colonies cultured was used to determine *Candida* species, colony numbers were used to calculate the number of CFU per milliliter. Clinical isolates were then stored on beads in glycerol in Microbank vials (Pro-Lab Diagnostics, Cheshire, UK) at −80°C. Each isolate was then subsequently identified by MALDI-TOF analysis using a Bruker Microflex system, comparing recorded spectra to the Bruker database to confirm identify to the species level.

*Candida* clinical isolates were assessed for biofilm-forming capabilities by crystal violet assay. *Candida* isolates (*n* = 33) were cultured on Sabouraud’s (SAB) agar for 48 h at 30°C. For biofilm formation, overnight cultures were grown in yeast extract peptone dextrose (YPD) at 30°C. Cultures were washed twice with phosphate-buffered saline (PBS) and standardized in RPMI 1640 medium to a final cell density of 1 × 10^6^ CFU/ml. Eight biofilms of each isolate were grown in 96-well, flat-bottomed polystyrene microtiter plates for 24 h at 37°C before biomass measured by the crystal violet assay (52). For visualization of *Candida* aggregates, 30 μl of CVL was stained with calcofluor white (CFW) to a final concentration of 0.06 μg/ml for 1 h at 37°C, before being imaged using the EVOS live cell imaging system (Thermo Scientific, Loughborough, UK).

### Antagonism of C. albicans and *Lactobacillus* in coculture.

The ability of the following 7 *Lactobacillus* strains to inhibit C. albicans SC5314 biofilm formation was assessed: *L. casei* ATCC 393, *L. fermentum* ATCC 14931, L. crispatus ATCC 33820, *L. iners* DSMZ 13335, *L. salivarius* ATCC 11741, L. jensenii ATCC 25258 and L. rhamnosus ATCC 7469. Overnight cultures of C. albicans and *Lactobacillus* were grown in YPD and de Man, Rogosa, and Sharpe (MRS; Merck UK) medium, respectively, under appropriate culture conditions. For these experiments, we used an optimized medium of Todd-Hewitt broth (THB; Merck UK) supplemented with 10 μM menadione and 10 μg/ml hemin (Thermo Fisher) and mixed 1:1 with RPMI (referred to here as 1:1 broth), as described previously for coculture experiments (53). For biofilm formation, overnight cultures were standardized to 1 × 10^6^ CFU/ml for C. albicans and 1 × 10^7^ CFU/ml for *Lactobacillus* species in 1:1 medium. Eight biofilms of each C. albicans-*Lactobacillus* pair were incubated in 5% CO_2_ for 24 h. In addition, C. albicans biofilms were grown for 4 h prior to *Lactobacillus* being added for 20 h, with biomass quantified using the crystal violet assay.

### C. albicans biofilm-related gene expression analysis.

To assess C. albicans biofilm-related gene expression, RNA was extracted from dual-species biofilms using the PureLink RNA minikit (Thermo Scientific, Loughborough, UK), following manufacturer’s instructions. In brief, 2 μg of RNA was converted to cDNA using a high-capacity cDNA reverse transcription kit (Thermo Scientific, Loughborough, UK) and 1 μl was used in a 20-μl qPCR with 10 μl 2× Fast SYBR green PCR master mix, 1 μl forward/reverse primer, and UV-treated H_2_O. Primer sequences can be found at https://www.glasgowbiofilms.co.uk/ (54–56). Gene expression was analyzed in duplicate on three separate occasions; no-reverse-transcription (NRT) controls and no-template controls (NTC) were included throughout. Gene expression was normalized to the *ACT1* housekeeping gene and calculated using the ΔΔ*C_T_* method (57).

### *In vitro* transcriptomic analysis of C. albicans interactions with L. crispatus.

C. albicans and L. crispatus type strains SC5314 and ATCC 33820, respectively, were used for transcriptional analysis in this component of the study. Biofilms were formed as described above. Cultures were washed twice with PBS and standardized in 1:1 broth to 1 × 10^6^ CFU/ml for C. albicans and 1 × 10^7^ CFU/ml for L. crispatus. Initially, C. albicans biofilms were grown in T-75 cell culture flasks (Corning, USA) for 4 h in 5% CO_2_. Following incubation, medium was removed, and biofilms washed before L. crispatus was added for an additional 2, 4, or 20 h. At each time point, the medium was removed and biofilms were washed with PBS before being scraped into 1 ml of RNAlater (Thermo Scientific, Loughborough, UK). Spent medium from biofilms was retained, and pH was monitored throughout the experiment. RNA was extracted from microbial biofilms using the RiboPure RNA purification kit for yeast (Thermo Scientific, Loughborough, UK), following manufacturer’s instructions. Integrity of RNA was assessed using a Bioanalyzer system, and genome-wide *Candida* transcripts were sequenced using the Illumina NOVASeq6000 sequencing platform (Edinburgh Genomics). FastQC was used to assign quality scores to the produced reads, and Illumina adaptors and poor-quality reads were trimmed using Trimmomatic (V0.38). Hisat2 (v2.1.0) was then used to align the resulting reads to a reference C. albicans genome (http://www.candidagenome.org/) before the number of sequences that were aligned to each gene was counted using HTSeq-count (v0.11.0). The counted genes were subsequently imported into RStudio, and the DESeq2 package was used to analyze the differentially expressed genes. A summarized illustration of the experimental and bioinformatics pipelines can be found in Fig. S1b and c.

### Investigating the probiotic potential of L. crispatus in a complex biofilm model.

Complex 11-species biofilms were formed as described previously by our group with slight modifications (58). Overnight broths of each organism were standardized in 1:1 medium prior to addition to the biofilm on 13-mm discs. For probiotic treatment, each biofilm was treated twice daily (12 h intervals) with 5 × 10^7^ CFU/ml of L. crispatus for 5 min before treatment was removed, biofilms washed 3 times in PBS and fresh media replaced. On day 3, after 4 probiotic treatments, 48-h biofilms were analyzed for compositional analysis. On day 5, following 8 probiotic treatments, 96-h biofilms were removed for compositional analysis. At each time point, biofilms were washed 3 times and sonicated in 1 ml of PBS at 35 kHz for 10 min to remove biomass. Sonicates were split in two, with one sample having 5 μl of 10 mM propidium monoazide (PMA) added to it for the quantification of live C. albicans DNA. PMA is a DNA-intercalating dye used to bind DNA from dead cells or those with a compromised membrane following exposure to a halogen light (59, 60). The other sample, lacking PMA, allowed quantification of total C. albicans DNA per biofilm. All samples were incubated in the dark for 10 min, then placed on ice, and exposed to a 650-W halogen light for 5 min. DNA was then extracted using the QIAamp DNA minikit, per the manufacturer’s instructions (Qiagen, Crawley, UK). To a 20-μl qPCR mixture, 1 μl of biofilm DNA was added; the mixture contained 10 μl Fast SYBR green master mix, 1 μl of 10 μM C. albicans forward and reverse primers, and UV-treated nuclease-free water. Primer sequences can be found at https://www.glasgowbiofilms.co.uk/. The following thermal profile was used: 95°C for 2 min and 40 cycles of 95°C for 3 s followed by 55°C for 30 s. Samples were assessed in duplicate from 2 separate experiments. Fungal CFE/ml were then calculated as described above.

### Statistical analysis.

Microbiome figures were created using MicrobiomeAnalyst (61). Bacterial diversity and abundance plots were made using alpha diversity and stacked-bar charts, respectively. Random forest plots were used to predict important species present under each condition. Transcriptome pipeline figures were constructed using BioRender and differential gene expression plots using the DESeq2 package in RStudio. Gene ontology networks were constructed using ClueGO software, available through Cytoscape (62). All other figures and analyses were performed in GraphPad Prism (version 8; GraphPad, La Jolla, CA, USA).

### Data availability.

Microbiome data are deposited under the accession number PRJNA719953 in the SRA database.
