# Supplementary figures and images for: Recurrent Vulvovaginal Candidiasis: a Dynamic Interkingdom Biofilm Disease of Candida and Lactobacillus
Source: mSystems. 2021 Aug 10;6(4):e00622-21. doi: 10.1128/mSystems.00622-21 (PMC8407231; doi:10.1128/mSystems.00622-21)

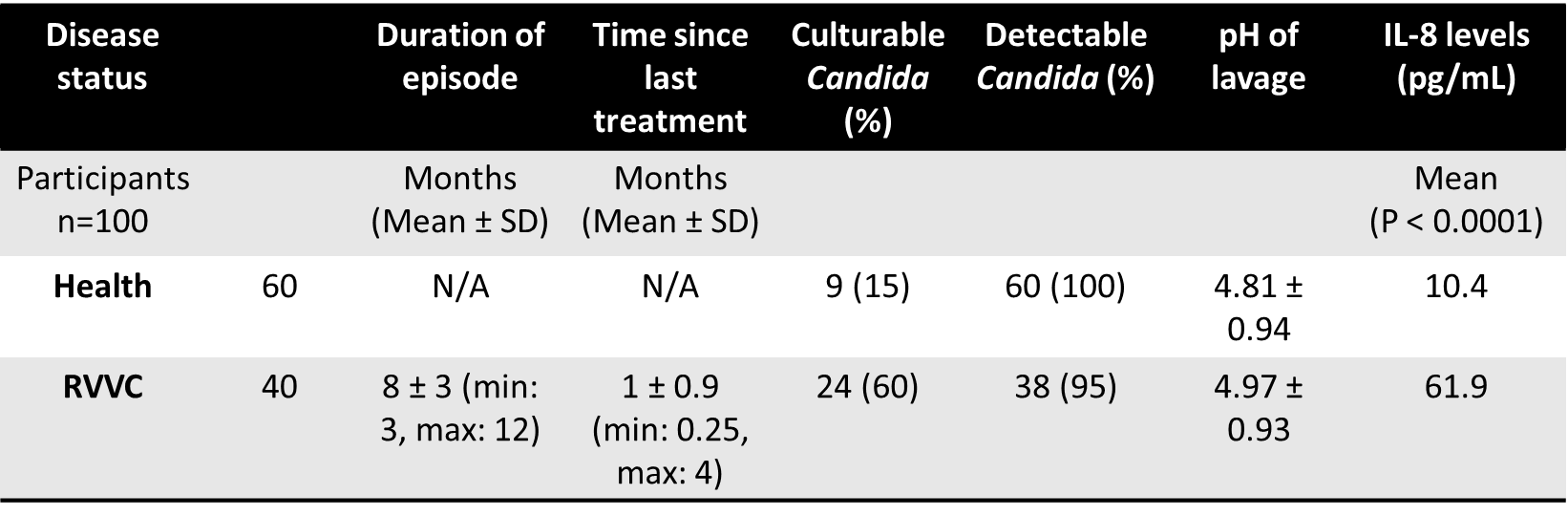

Supplement: TABLE S1 [file msystems.00622-21-st001.docx]

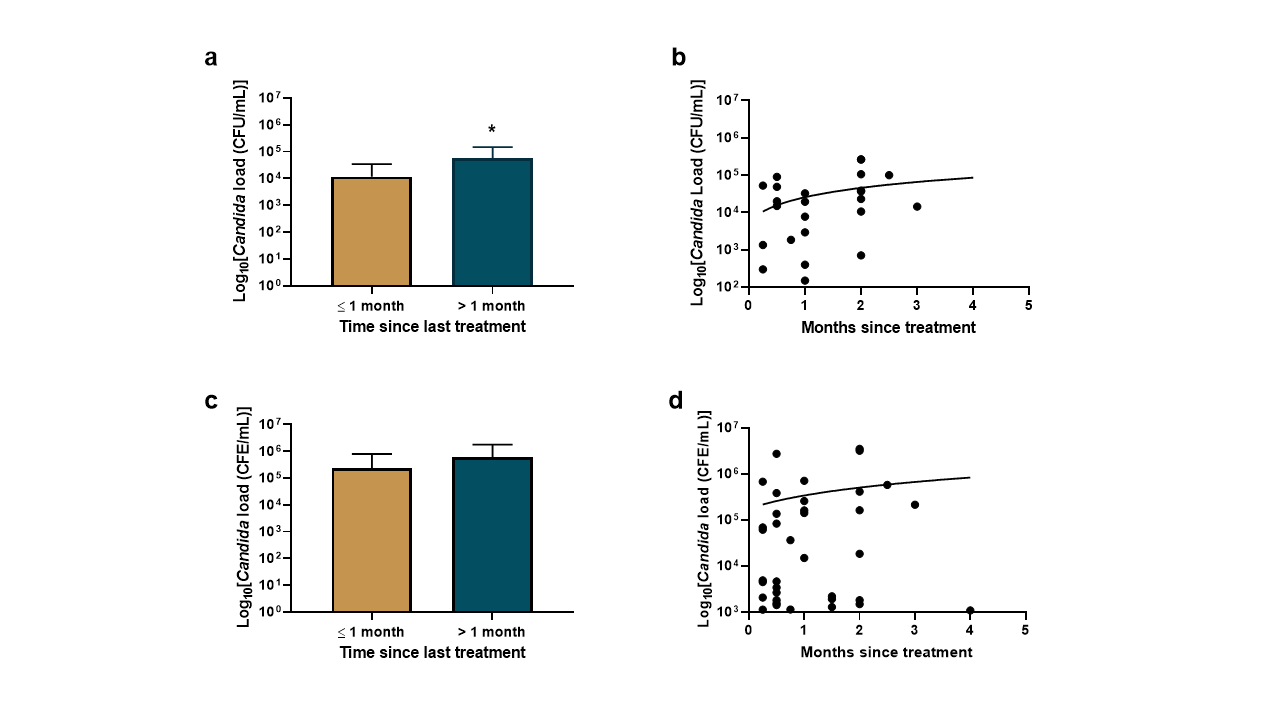

Supplement: FIG S2 [file msystems.00622-21-sf002.tif]

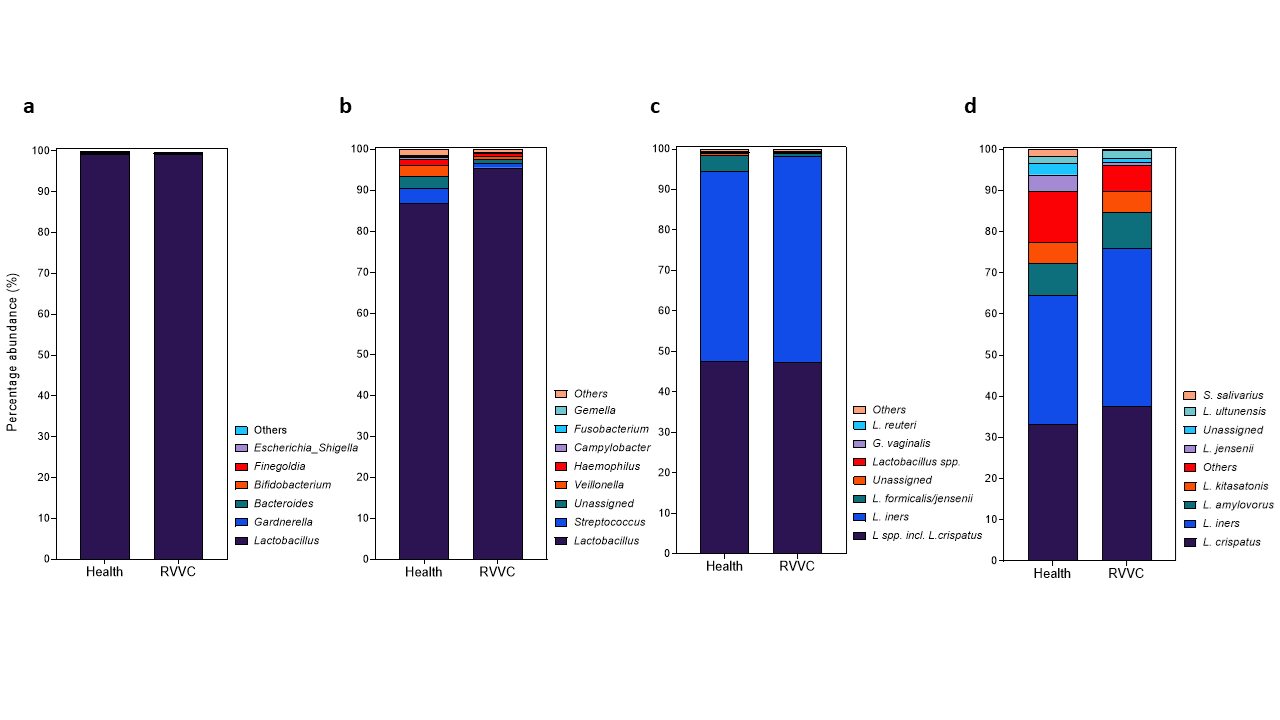

Supplement: FIG S3 [file msystems.00622-21-sf003.tif]

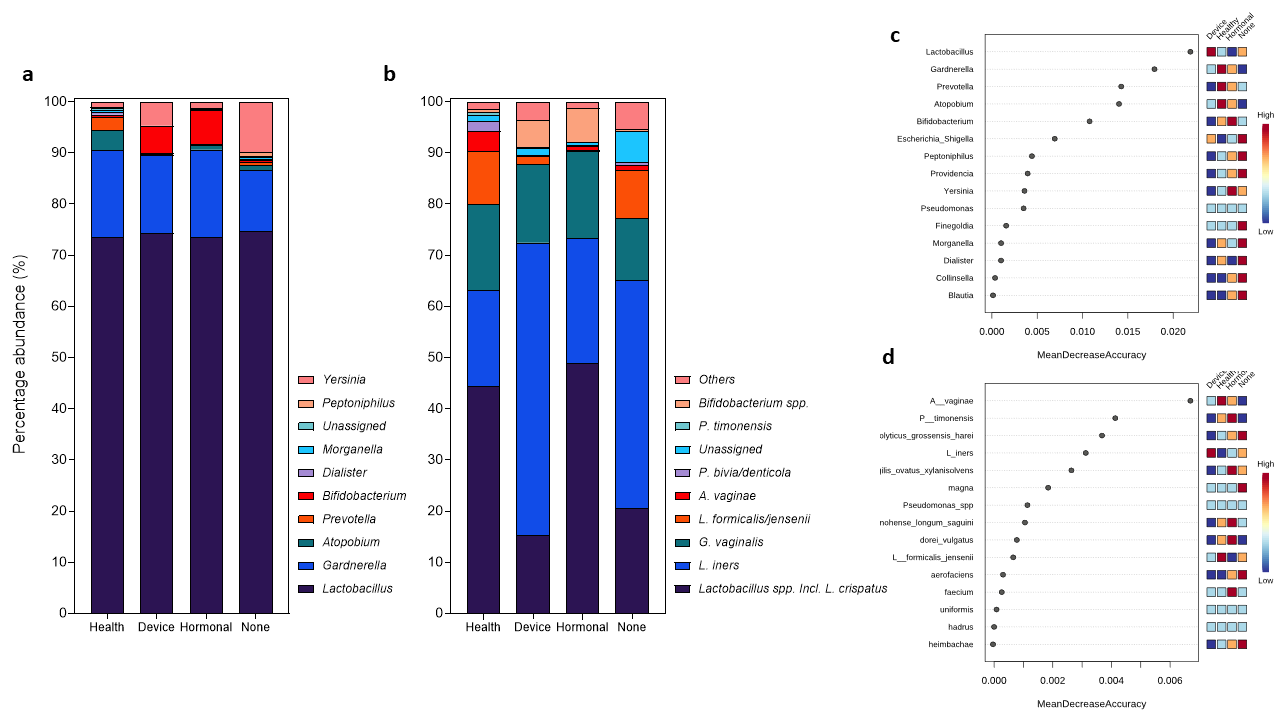

Supplement: FIG S4 [file msystems.00622-21-sf004.tif]

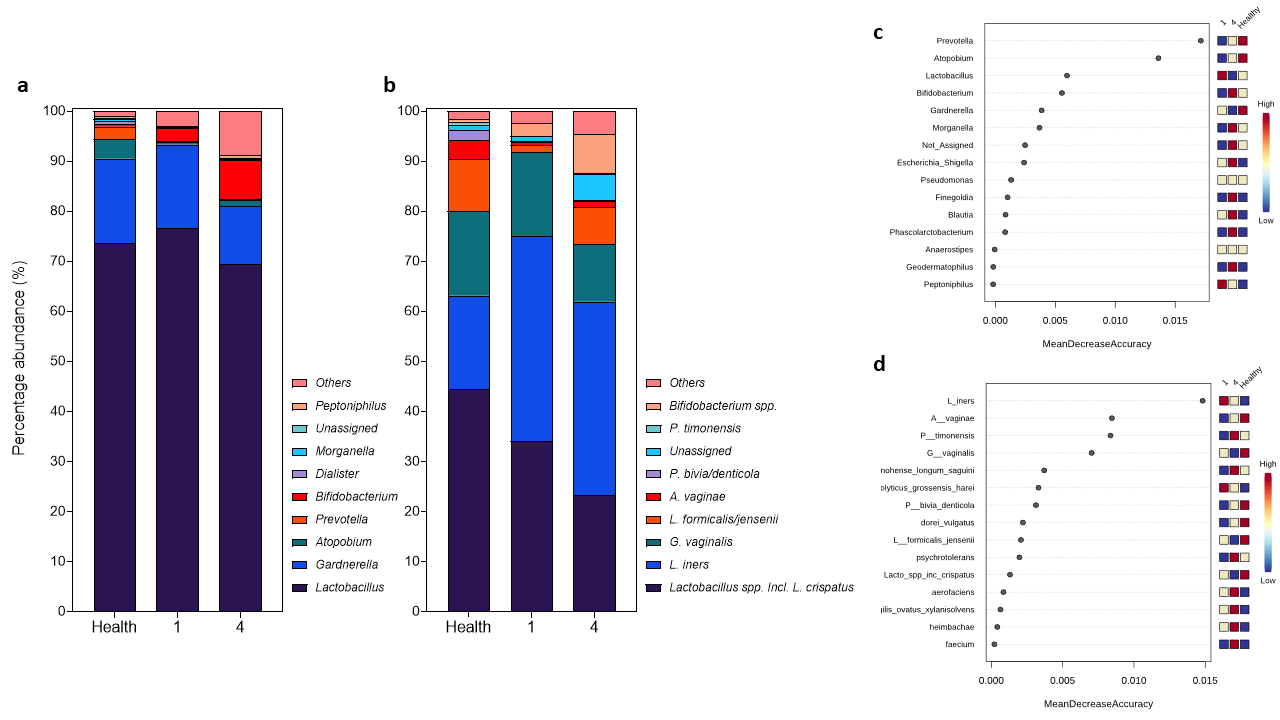

Supplement: FIG S5 [file msystems.00622-21-sf005.tif]

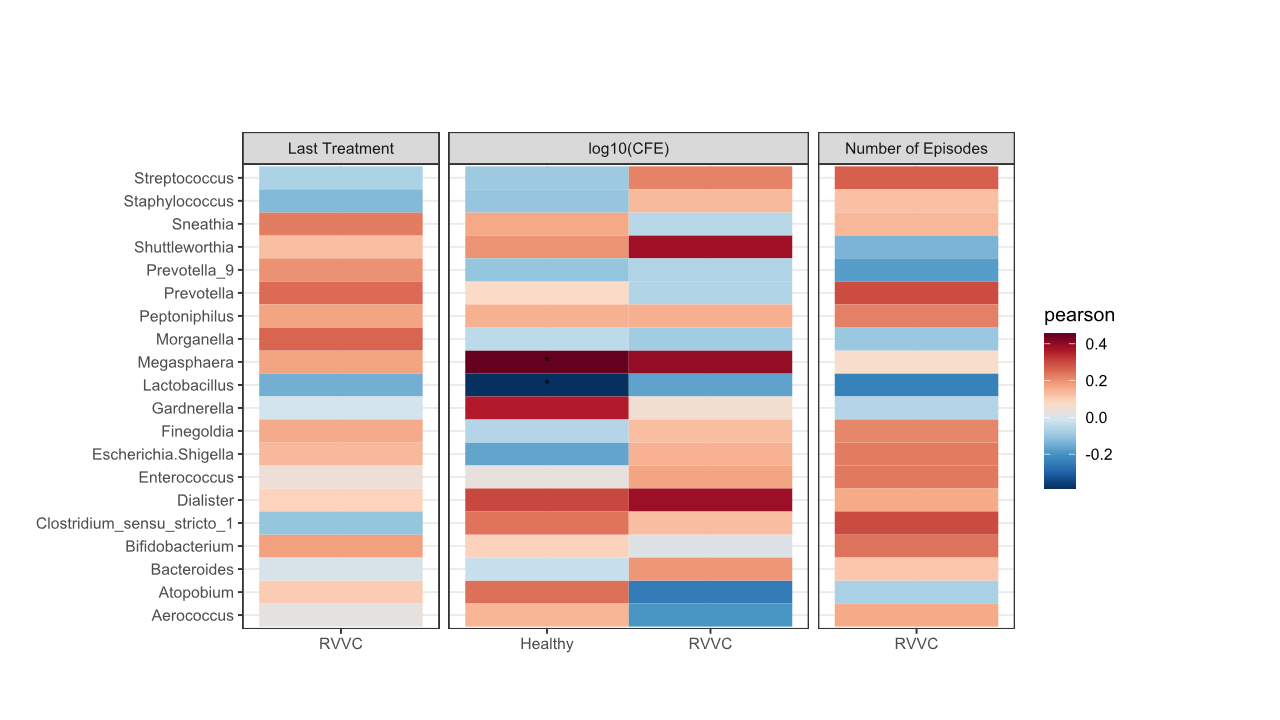

Supplement: FIG S6 [file msystems.00622-21-sf006.tif]

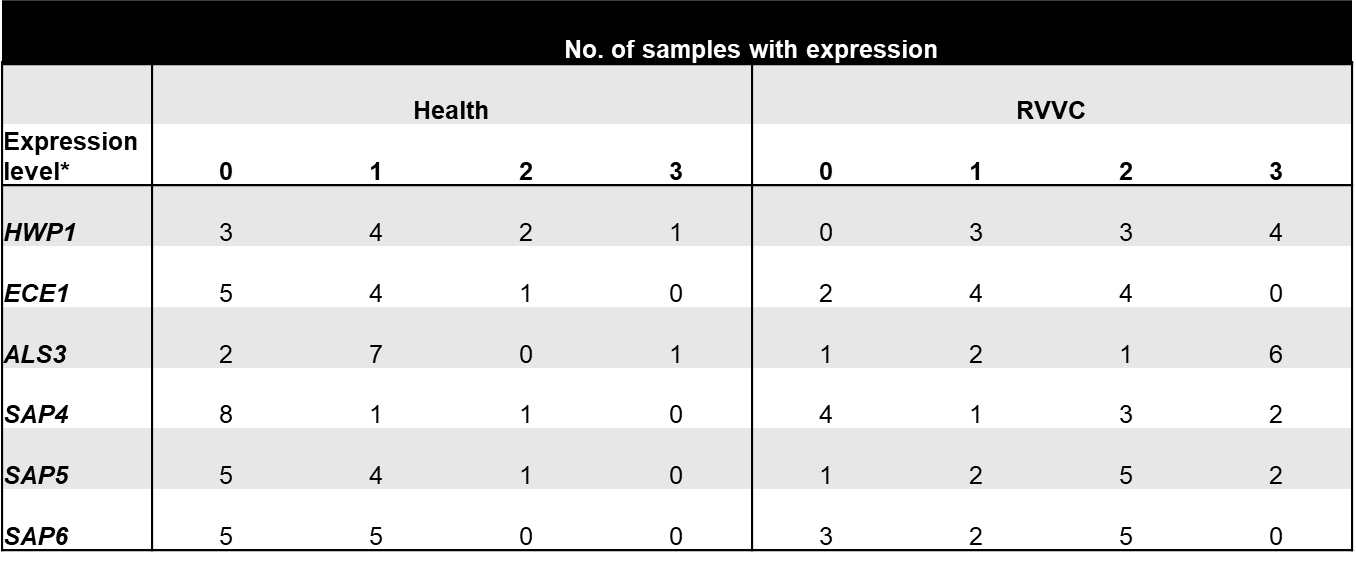

Supplement: TABLE S2 [file msystems.00622-21-st002.docx]

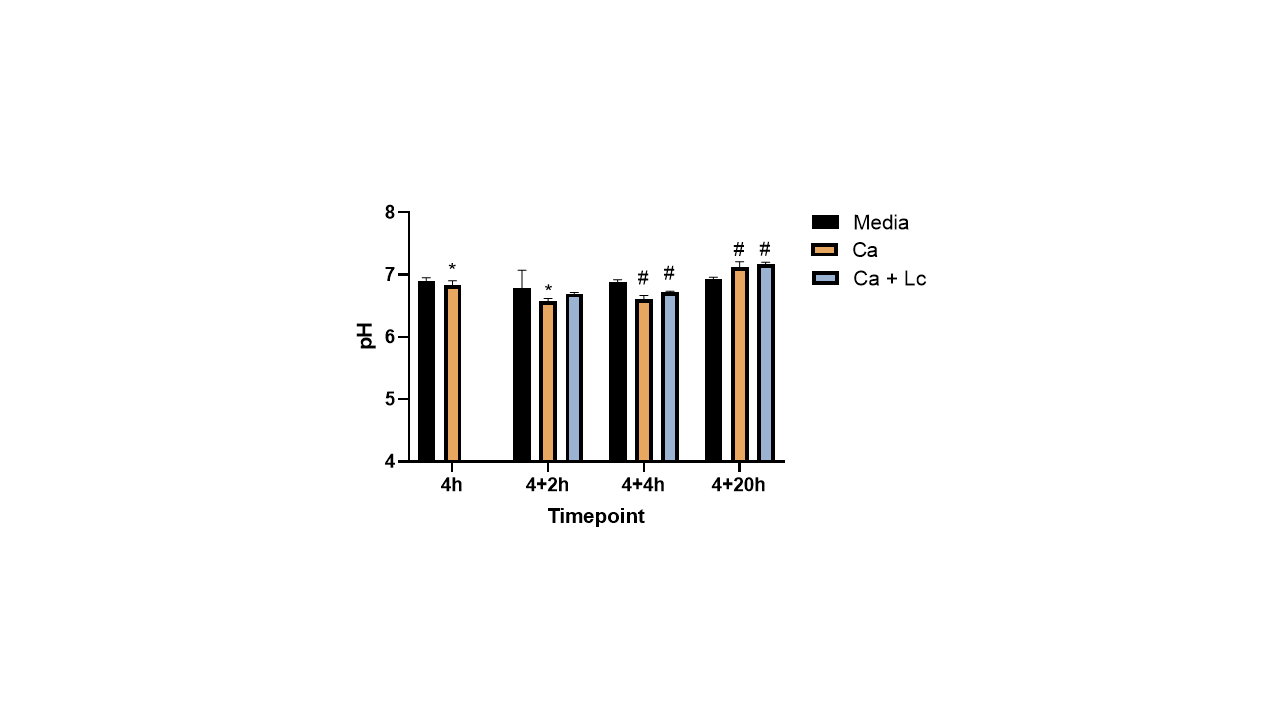

Supplement: FIG S7 [file msystems.00622-21-sf007.tif]

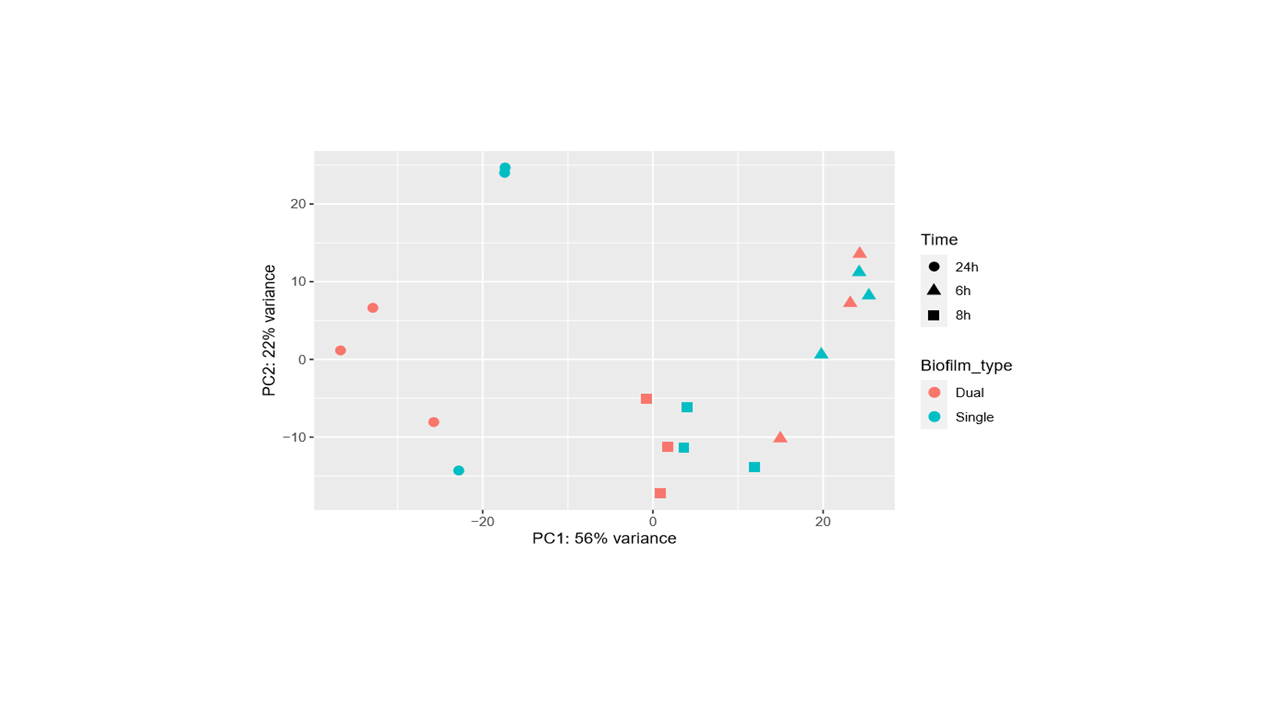

Supplement: FIG S8 [file msystems.00622-21-sf008.tif]

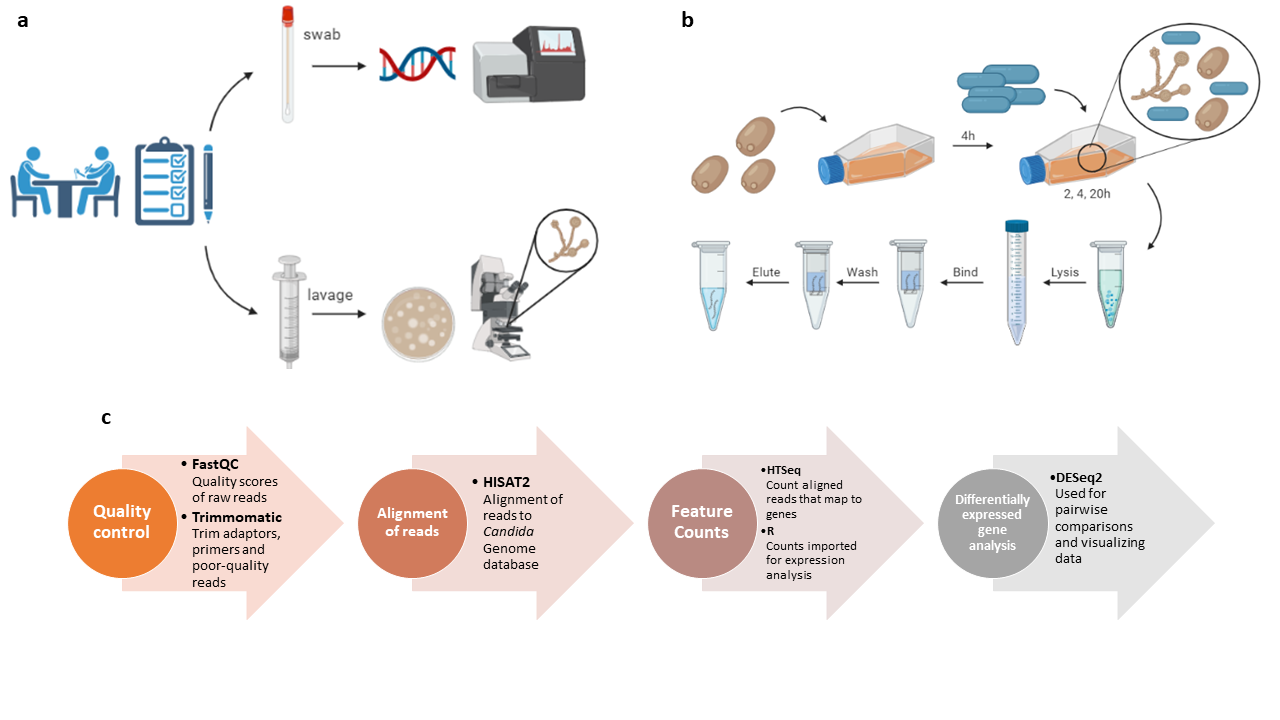

Supplement: FIG S1 [file msystems.00622-21-sf001.tif]
